# Fibrosis-associated hepatocarcinogenesis revisited: Establishing standard medium-term chemically-induced male and female models

**DOI:** 10.1371/journal.pone.0203879

**Published:** 2018-09-13

**Authors:** Guilherme Ribeiro Romualdo, Gabriel Bacil Prata, Tereza Cristina da Silva, Ana Angélica Henrique Fernandes, Fernando Salvador Moreno, Bruno Cogliati, Luís Fernando Barbisan

**Affiliations:** 1 Department of Pathology, Botucatu Medical School, São Paulo State University (UNESP), Botucatu, São Paulo, Brazil; 2 Department of Morphology, Biosciences Institute, São Paulo State University (UNESP), Botucatu, São Paulo, Brazil; 3 Department of Pathology, School of Veterinary Medicine and Animal Science, University of São Paulo (USP), São Paulo, São Paulo, Brazil; 4 Department of Chemistry and Biochemistry, Biosciences Institute, São Paulo State University (UNESP), Botucatu, São Paulo, Brazil; 5 Department of Food and Experimental Nutrition, Faculty of Pharmaceutical Sciences, University of São Paulo (USP), São Paulo, São Paulo, Brazil; IDIBAPS Biomedical Research Institute, SPAIN

## Abstract

Hepatocellular carcinoma causes ~10% of all cancer-related deaths worldwide, usually emerging in a background of liver fibrosis/cirrhosis (70%-90% of cases). Chemically-induced mouse models for fibrosis-associated hepatocarcinogenesis are widely-applied, resembling the corresponding human disease. Nonetheless, a long time is necessary for the development of preneoplastic/neoplastic lesions. Thus, we proposed an early fibrosis-associated hepatocarcinogenesis model for male and female mice separately, focusing on reducing the experimental time for preneoplastic/neoplastic lesions development and establishing standard models for both sexes. Then, two-week old susceptible C3H/HeJ male and female mice (n = 8 animals/sex/group) received a single dose of diethylnitrosamine (DEN, 10 or 50 mg/Kg). During 2 months, mice received 3 weekly doses of carbon tetrachloride (CCl_4_, 10% corn oil solution, 0.25 to 1.50 μL/g b.wt.) and they were euthanized at week 17. DEN/CCl_4_ protocols for males and females displayed clear liver fibrosis, featuring collagen accumulation and hepatic stellate cell activation (α-SMA). In addition, liver from males displayed increased CD68+ macrophage number, COX-2 protein expression and IL-6 levels. The DEN/CCl_4_ models in both sexes impaired antioxidant defense as well as enhanced hepatocyte proliferation and apoptosis. Moreover, DEN/CCl_4_-treated male and female developed multiple preneoplastic altered hepatocyte foci and hepatocellular adenomas. As expected, the models showed clear male bias. Therefore, we established standard and suitable fibrosis-associated hepatocarcinogenesis models for male and female mice, shortening the experimental time for the development of hepatocellular preneoplastic/neoplastic lesions in comparison to other classical models.

## Introduction

Hepatocellular carcinoma (HCC), the main type of primary liver cancer, is responsible for ~10% of all cancer-related deaths worldwide (~800,000 deaths/year, considering both genders) [1]. Although epidemiological data presents clear gender disparity, assuming that HCC is about three times more common in men (554,000 *vs*. 228,000 new cases/year), this malignant neoplasia displays increasing importance in women, being the ninth most common cancer and the sixth leading cause of cancer-related deaths in this gender [1].

HCC is considered a complex, multistep and multifactorial disease and it usually emerges in a background of liver fibrosis/cirrhosis (70% to 90% of all HCC cases), mainly caused by chronic hepatitis B and C virus infections, chronic ethanol abuse and nonalcoholic steatohepatitis (NASH) [2,3]. The pro-inflammatory and pro-fibrotic environment provided by these risk factors are the necessary background for the emerging of genetic and epigenetic alterations that can promote the development of dysplastic nodules and neoplastic lesions, mainly HCC [4].

The establishment of chemically-induced hepatocarcinogenesis models in rodents proved to be essential for both basic and translational research because of their remarkable similarities to the corresponding human disease [5,6]. Nonetheless, due to the great variability of chemically-induced protocols (*e*.*g*. different chemical compounds, doses, frequencies of administration, etc.) and mouse strains used (less or more susceptible), the literature lacks standard mice models for hepatocarcinogenesis in males and especially in females, neglected because the models reflect the HCC male bias in humans [7]. In addition, a long latency time is necessary for the development of hepatocellular preneoplastic and/or neoplastic lesions in these models [5]. Finally, most of chemically-induced hepatocarcinogenesis mice models do not feature liver fibrosis, calling into question whether these models can reliably recapitulate key events observed during human hepatocarcinogenesis and HCC progression.

In this context, this study aimed at proposing a medium-term fibrosis-associated hepatocarcinogenesis mouse model for male and female mice separately, allying chemically-induced protocol and a susceptible mouse strain in order to reduce the experimental time to the development of hepatocellular preneoplastic and neoplastic lesions associated to liver fibrosis. Thus, the establishment of standard medium-term mice models may favor the emerging diagnostic, preventive and therapeutic methods for fibrosis-associated hepatocarcinogenesis in humans.

## Materials and methods

### Experimental design

**Fig 1:**
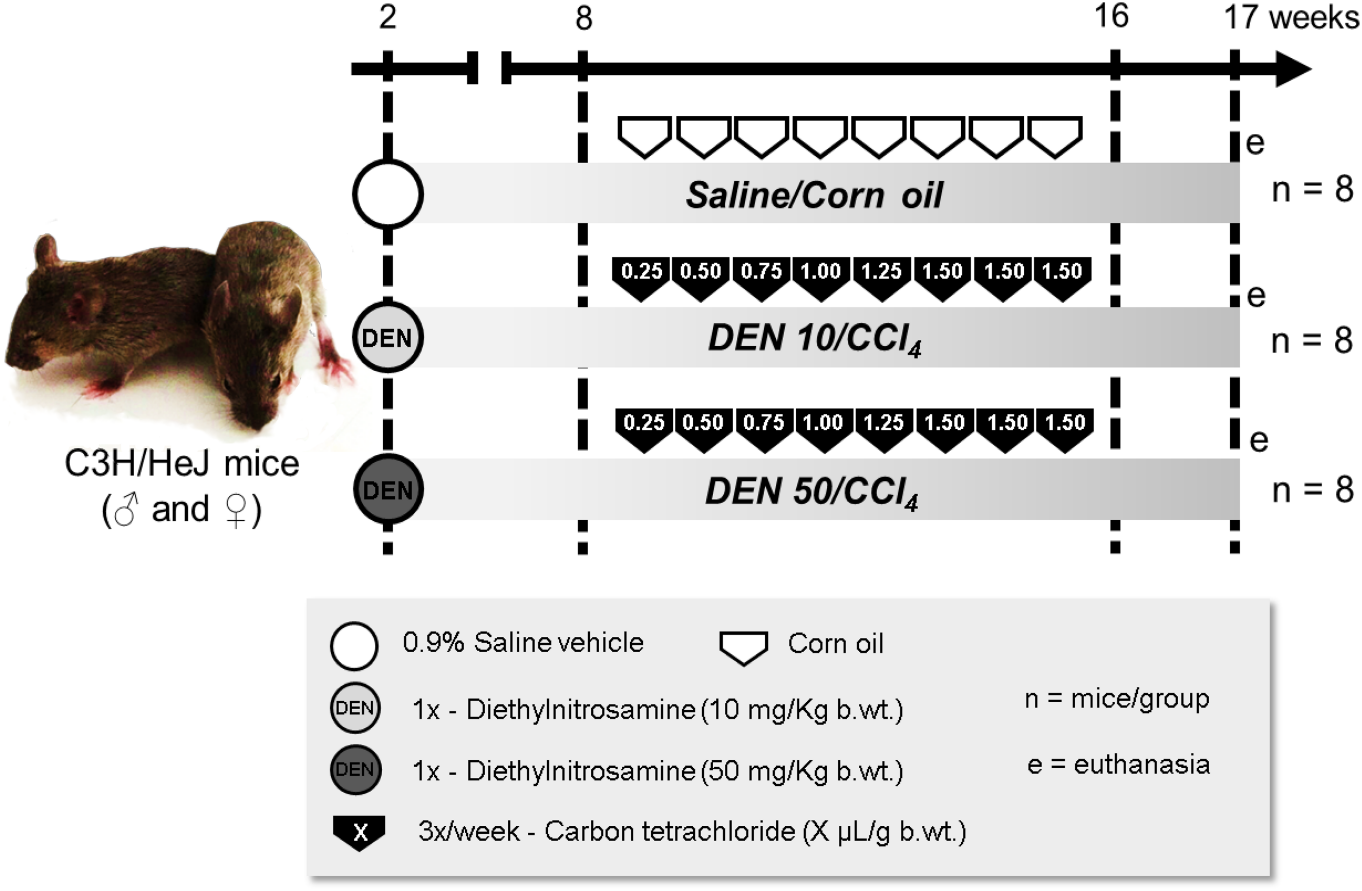
Experimental design. For details, see “Materials and methods” section.

In order to reduce the experimental period, the C3H/HeJ mouse strain was selected due to its increased susceptibility to DEN-induced hepatocarcinogenesis compared to Balb/C and C57BL mouse strains [8,9]. C3H/HeJ male and female mice (n = 8 animals/sex/group), obtained from the Department of Pathology of School of Veterinary Medicine and Animal Science (University of São Paulo) and kept in Botucatu Medical School (São Paulo State University), were submitted to a classical infant hepatocarcinogenesis model [10] by receiving a single intraperitoneal (i.p.) injection of diethylnitrosamine [DEN, 10 or 50 mg/kg body weight (b.wt.) in 0.9% saline, Sigma-Aldrich, USA] at postnatal day (PND) 14 or just saline vehicle (Fig 1). Both single 10 and 50 mg/Kg DEN doses were based on previous studies [11,12]. Mice were weaned at PND 28 (week 4). From week 8 to 16, in order to promote DEN-induced hepatocellular preneoplastic and neoplastic lesions in a background of liver fibrosis, mice received 3 weekly i.p. doses of carbon tetrachloride (CCl_4_, 10% solution in corn oil, Sigma-Aldrich, USA) or just vehicle (Fig 1). The initial dose of CCl_4_ was 0.25 μL/g b.wt., and there were 0.25 μL weekly increments to the utmost dose of 1.50 μL/g b.wt. [13]. All animals were euthanized by exsanguination under isoflurane inhalant anesthesia at week 17, a week after the last CCl_4_ administration (Fig 1). Blood was collected from cava vein into heparinized syringes and centrifuged for 10 minutes at 1503*xg*, and samples were stored at -80°C for further analysis. Liver was sampled for histological analysis and additional samples were collected, snap-frozen in liquid nitrogen and stored at -80°C. All animals were kept in propylene cages with stainless steel cover and pine wood shavings bedding in a room with continuous ventilation (16–18 air changes/hour), relative humidity (45–65%), controlled temperature (20–24°C) and light/dark cycle 12:12 and were given drinking water and chow (Nuvital—Nuvilab, Brazil) *ad libitum*. Body weight and food consumption, as well as the health condition of the animals, were monitored and recorded one time *per* week during all the experimental period. The animal experiment was carried out under protocols approved by Botucatu Medical School/UNESP Ethics Committee on Use of Animals (CEUA) (Protocol number 1186/2016). All animals received humane care according to the criteria outlined in the “Guide for the Care and Use of Laboratory Animals” [14].

### Macroscopy, histopathology and collagen morphometric evaluation

At necropsy, the liver was removed, weighed, washed in saline solution (0.9% NaCl) and grossly examined for the occurrence of liver nodules [15]. All macroscopically visible liver nodules greater than 1 mm in diameter were registered. The incidence and multiplicity of the alterations were calculated for each group. Then, samples were collected from “normal-appearing” liver tissue (left lateral, right medial and caudate lobes) and from macroscopic gross lesions (for further histological diagnosis). The samples were fixed in 10% buffered formalin for 24 h at room temperature, stored in 70% ethanol and embedded in paraffin wax. Five-micron thick liver sections from paraffin-embedded blocks were obtained and stained with Hematoxylin and Eosin (HE) and Sirius Red.

Preneoplastic and neoplastic lesions were identified in HE-stained sections using previously well-established criteria [16] and their incidences were calculated for each group. For hepatocellular adenoma (HCA), it was also calculated the multiplicity (number of lesions/mice). For preneoplastic altered hepatocyte foci (AHF), it was calculated *(1) AHF number per liver area*, counting all AHF and dividing by the section liver area analyzed, *(2) AHF size*, by individually measuring all AHF, and *(3) AHF area*, dividing the sum of all AHF areas by the liver section area analyzed. The liver section area (cm^2^) was measured by Stemi 2000 stereo zoom microscope (Zeiss, Germany) using a Dino Capture (ANMO Electronics Corporations, USA) image analysis system. The AHF size (mm^2^) was measured by Olympus CellSens software (Olympus Corporation, Japan). The morphometric analysis of collagen in Sirius red-stained sections was performed according to previous studies [13] using Image-Pro Plus 4.5 software (Media Cybernetics, USA) in 10 randomly selected fields (20× objective) *per* section (left lobe), comprising portal areas [Collagen area (%) = Sirius red area / (total field area − vascular luminal area)].

### Immunohistochemistry and semi-quantitative analysis

Immunoreactivity for α-smooth muscle actin (α-SMA, *i*.*e*. activated HSC marker), CD68 (*i*.*e*. macrophage/Kupffer cell marker), Ki-67 (*i*.*e*. cell proliferation marker), cleaved caspase-3 (*i*.*e*. apoptosis marker), transforming growth factor-α (TGF-α), cyclooxygenase-2 (COX-2) and β-catenin were detected using a one-step polymer-HRP system (EasyPath—Erviegas, Brazil). Briefly, deparaffinated 5-μm liver sections on silane-covered microscope slides were subjected to antigen retrieval in 0.01M citrate buffer (pH 6.0) at 120°C for 5 min in a Pascal Pressure Chamber (Dako Cytomation, Denmark). After endogenous peroxidase blockade with 1% H_2_O_2_ in phosphate-buffered saline (PBS) for 15 minutes, the slides were treated with low-fat milk for 60 min and incubated in a humidified chamber overnight at 4°C with anti-α-SMA (ab124964, 1:500 dilution, Abcam, UK), anti-CD68 (ab125212, 1:1000 dilution, Abcam, UK), anti-Ki-67 (ab16667, 1:100 dilution, Abcam, UK), anti-cleaved caspase-3 (5A1E, 1:50 dilution, Cell Signaling, USA), anti-β-catenin (ab32572, 1:400 dilution, Abcam, UK), anti-TGF-α (ab9585, 1:500 dilution, Abcam, UK) or anti-COX-2 (SP21, 72 kDa, 1:100 dillution, Biocare Medical, USA) primary antibodies. Then, the slides were incubated with a one-step polymer-HRP for 20 minutes. The reaction was visualized with 3´3-diaminobenzidine (DAB) chromogen (Sigma–Aldrich, USA) and counterstained with Harris hematoxylin.

For Ki-67, cleaved caspase-3 and CD68 semi-quantitative analysis in surrounding liver tissue (avoiding preneoplastic and neoplastic lesions), 10 random fields (40× objective) were assessed in left hepatic lobe sections. The Ki-67- and cleaved caspase-3-positive hepatocytes and CD68-positive macrophages were counted and divided by the liver area analyzed (mm^2^). In AHF and HCAs, all Ki-67-positive hepatocytes were counted and divided by AHF or HCA area (mm^2^). We also analyzed the TGF-α phenotype in AHF, calculating the incidence of TGF-α positive foci [17]. For β-catenin, the cellular location (membrane, cytoplasm or and/or nucleus) was evaluated in the hepatocytes of surrounding liver tissue, FHA and HCA [12]. These analyses were performed in Olympus CellSens software (Olympus Corporation, Japan). The α-SMA semi-quantitative analysis was performed using Image-Pro Plus 4.5 software (Media Cybernetics, USA) in 10 randomly selected fields (20× objective) *per* section (left lobe), comprising portal areas [α-SMA area (%) = α-SMA positive area / (total field area − vascular luminal area)].

### Western blot

Liver samples from the left lobe (~100 mg) were homogenized in lysis buffer (500 nM Tris-HCl, 0.2 M NaCl, 0.1% Triton X-100, 10 mM CaCl_2_, and 10 μl/mL protease inhibitor cocktail [Sigma-Aldrich, USA]) in the proportion of 30 mg of tissue/100 μl of buffer (4°C, 2 h). After this procedure, the extracted material was centrifuged (1500×g, 4°C, 20 min) and the supernatant collected for protein quantification by Bradford method. Aliquots of liver homogenates containing 7 μg of total protein were heated (95°C, 5 min) in Laemmli sample buffer (2.5 mM Tris, 2% SDS, 10% glycerol, 0.01% bromophenol blue, 5% 2-mercaptoethanol) and then electrophoretically separated in a 10% SDS–PAGE gel under reducing conditions and transferred to nitrocellulose membranes (Sigma Chemical, USA). Membranes were blocked with non-fat milk in TBS-T (1 M Tris, 5 M NaCl, pH 7.2, 500 μL Tween-20) (1 h).

Membranes were subsequently incubated with rabbit polyclonal anti-NFκB p65 (sc-372, 65 kDa, 1:1000 dilution, Santa Cruz Biotechnology, USA), rabbit monoclonal anti-β-catenin (ab32572, 92 kDa, 1:7000 dilution, Abcam, UK), anti-COX-2 (SP21, 72 kDa, 1:1000 dillution, Biocare Medical, USA) or goat polyclonal anti-actin (sc1815, 43 KDa, 1:1000 dilution, Santa Cruz Biotechnology, USA) primary antibodies in 5% BSA solution overnight. After 5 wash steps with TBS-T, membranes were incubated with specific horseradish conjugated secondary antibodies, according to the primary antibodies used (2 h). Finally, after 5 wash steps, the membranes were submitted to immunoreactive protein signals detected using an Amersham ECL Select Western Blotting Detection Reagent (GE Healthcare Life Sciences, UK). Signals were captured by a G:BOX Chemi system (Syngene, UK) controlled by an automatic software (GeneSys, Syngene, UK). Band intensities were quantified using densitometry analysis Image J software (National Institutes of Health, USA). Finally, NFkB, COX-2 and β-catenin, protein expression were reported as fold change according to β-actin protein expression used as a normalizer. For each marker, we ran 2 samples of each group/gel, totalizing 3 gels and 6 samples/group.

### Interleukin-6 (IL-6) analysis

Liver samples (~100 mg) were homogenized in phosphate buffer, centrifuged (10000×g, 4°C, 35 min) and IL-6 protein levels were evaluated by enzyme-linked immunosorbent assay (ELISA) in homogenate using Mouse IL-6 DuoSet ELISA (R&D Systems, USA). The assays were performed according to the manufacturer’s instructions. The detection limit of IL-6 was found to be 2500–19.5 pg/mL.

### Serum transaminases and liver biochemistry

Serum alanine (ALT) and aspartate (AST) aminotransferase levels were determined by a conventional kinetic assay according to the manufacturer’s instructions (Liquiform Labtest Diagnóstica, Brazil). These determinations were performed in an automated spectrophotometric (Labmax 240 analyzer, Labtest Diagnóstica, Brazil).

For biochemical analysis, liver samples from the left lobe were homogenized in 50 mM phosphate buffer (pH 7.4) using a motor–driven Teflon glass Potter Elvehjem (100×g/min) and centrifuged (12000×g, -4°C, 15 min). The supernatant was used to determine catalase, superoxide dismutase (SOD), glutathione peroxidase (GSH-Px) activities and total glutathione, reduced glutathione (GSH) and lipid hydroperoxide levels. Catalase activity was determined in sodium and potassium phosphate buffer containing 10 mM hydrogen peroxide [18]. GSH-Px activity was assayed by following the oxidation of 0.16 mM NADPH in the presence of glutathione reductase which catalyzed the reduction of oxidized glutathione (GSSG) formed by GSH-Px [19]. SOD activity was determined based on the ability of the enzyme to inhibit the reduction of nitro blue tetrazolium (NBT), which was generated by hydroxylamine in a medium containing phosphate buffer, 0.1 mM EDTA, 50 mM NTB, 78 mM NADH and 33 mM phenazine methosulfate [20]. GSH was measured in medium containing 2 mM 5,5′-dithiobis-(2-nitrobenzoic) acid (DTNB, Sigma-Aldrich, USA), 0.2 mM NADPH, and 2 U of glutathione reductase in phosphate buffer (100 mM, pH 7.4) 5 mM EDTA [21]. The total glutathione was assayed with 0.6 mM DTNB, and 1 U of glutathione reductase in buffer 0.1 M Tris–HCl, pH 8.0 containing 0.5 mM EDTA [21]. The lipid hydroperoxide levels were measured through hydroperoxide-mediated oxidation of Fe^2+^, with 100 μL of sample and 900 μL of a reaction mixture containing 250 μM FeSO_4_, 25 mM H_2_SO_4_, 100 μM xylenol orange and 4 mM butylated hydroxytoluene in 90% (v/v) methanol [22]. All determinations were performed using a microplate reader (25°C) (mQuant-Gen5 2.0 software, Bio-Tec Instruments, USA).

### Statistical analysis

When compared Saline/Corn oil, DEN 10/CCl_4_ and DEN 50/CCl_4_ groups, data were analyzed by one-way ANOVA or Kruskal-Wallis test followed by *post hoc* Tukey test or Dunn’s method, respectively. For DEN 10/CCl_4_ *vs*. DEN 50/CCl_4_ or male *vs*. female group comparisons, data were analyzed using Student t or Mann-Whitney tests. All data on incidence were analyzed by Fisher’s exact test. Differences were considered significant when p<0.05. Statistical analysis were performed using GraphPad Prism software (GraphPad, USA). All data were expressed as mean ± standard deviation of the mean (S.D.) or median and interquartile (q1-q3) (box-plots).

## Results

### General findings

Male mice submitted to DEN 50/CCl_4_ protocol showed a reduction in body weight throughout the experimental period (S1 Fig), but mainly at weaning (week 3, after DEN administration) (p<0.001), CCl_4_ onset (week 8) (p = 0.003) and at the end of the experiment (week 17) (p = 0.038) compared to vehicles and/or DEN 10/CCl_4_ groups (Table 1). In addition, DEN 50/CCl_4_-treated males presented a reduction in food intake when compared to the other groups (p<0.001) (Table 1). As reported previously, reduced body weight and food consumption are common features of drug-induced liver toxicity in rodents [6]. However, animals did not show severe signs of illness and no mortality was observed.

**Table 1:** Effects of different DEN/CCl_4_ protocols on body and liver weights, food consumption and serum transaminases levels of male and female C3H/HeJ mice.

Parameters	Groups^1^		
	Saline/Corn oil	DEN 10/CCl_4_	DEN 50/CCl_4_
*Males*			
Body weight at week 3 (weaning) (g)	9.89 ± 2.00^a^	9.12 ± 1.01^a^	7.42 ± 1.24^b^
Body weight at week 8 (CCl_4_ onset) (g)	23.27 ± 2.42^a^	22.19 ± 1.65^ab^	20.77 ± 1.08^b^
Final body weight (g)	27.92 ± 2.64^a^	27.15 ± 1.21^ab^	26.09 ± 1.01^b^
Food consumption (g/mouse/day)	4.34 ± 0.56^a^	4.60 ± 0.57^a^	3.76 ± 0.36^b^
Absolute liver weight (g)	1.39 ± 0.18^b^	1.80 ± 0.28^a^	1.56 ± 0.17^b^
Relative liver weight (%)	4.98 ± 0.49^b^	6.62 ± 0.97^a^	6.00 ± 0.57^a^
ALT (U/L)	32.90 ± 7.30^b^	63.40 ± 10.89^a^	62.55 ± 17.75^a^
AST (U/L)	47.20 ± 7.55^b^	67.40 ± 11.92^a^	59.46 ± 9.82^a^
*Females*			
Body weight at week 3 (weaning) (g)	9.08 ± 2.22^a^	7.69 ± 1.05^ab^	7.26 ± 1.33^b^
Body weight at week 8 (CCl_4_ onset) (g)	18.27 ± 2.31	18.06 ± 0.98	17.72 ± 1.42
Final body weight (g)	20.43 ± 2.56	21.46 ± 1.34	21.67 ± 1.31
Food consumption (g/mouse/day)	3.71 ± 0.31^b^	4.44 ± 0.33^a^	3.71 ± 0.26^b^
Absolute liver weight (g)	0.99 ± 0.13^b^	1.28 ± 0.18^a^	1.33 ± 0.27^a^
Relative liver weight (%)	4.88 ± 0.42^b^	5.99 ± 0.70^a^	6.13 ± 1.00^a^
ALT (U/L)	27.40 ± 5.58^b^	45.57 ± 11.85^a^	40.90 ± 4.17^a^
AST (U/L)	53.55 ± 6.87^b^	62.20 ± 9.44^ab^	64.60 ± 9.44^a^

Values are mean ± S.D. n = 8 mice/sex/group.

^1^DEN 10 or 50 = diethylnitrosamine 10 or 50 mg/kg b.wt. in 0.9% saline at week 2 and CCl_4_ = carbon tetrachloride, i.p., 0.25 to 1.50 μL/g b.wt., in 10% corn oil solution for 8 weeks (see Material and methods section). Different letters correspond to statistical difference among groups by ANOVA and *post hoc* Tukey’s test (p<0.05).

DEN 50/CCl_4_-treated females presented reduced body weight compared to vehicle-treated group only at weaning (week 3) (p = 0.046) (S1 Fig; Table 1). Although DEN 10/CCl_4_-treated females showed increased food consumption (p<0.001) (Table 1), all groups showed similar body weight evolution during the whole experiment (S1 Fig).

### Serum transaminases and liver macroscopy

In males, both DEN/CCl_4_ protocols displayed enhanced hepatocellular damage, indicated by an increase in serum ALT and AST levels compared to vehicle-treated group (p<0.001 and p = 0.003, respectively) (Table 1). The females submitted to DEN 10/CCl_4_ protocol only showed enhanced ALT levels (p<0.001) (Table 1), while DEN 50/CCl_4_ protocol increased both ALT and AST serum levels (p<0.001 and p = 0.005, respectively) (Table 1).

**Fig 2:**
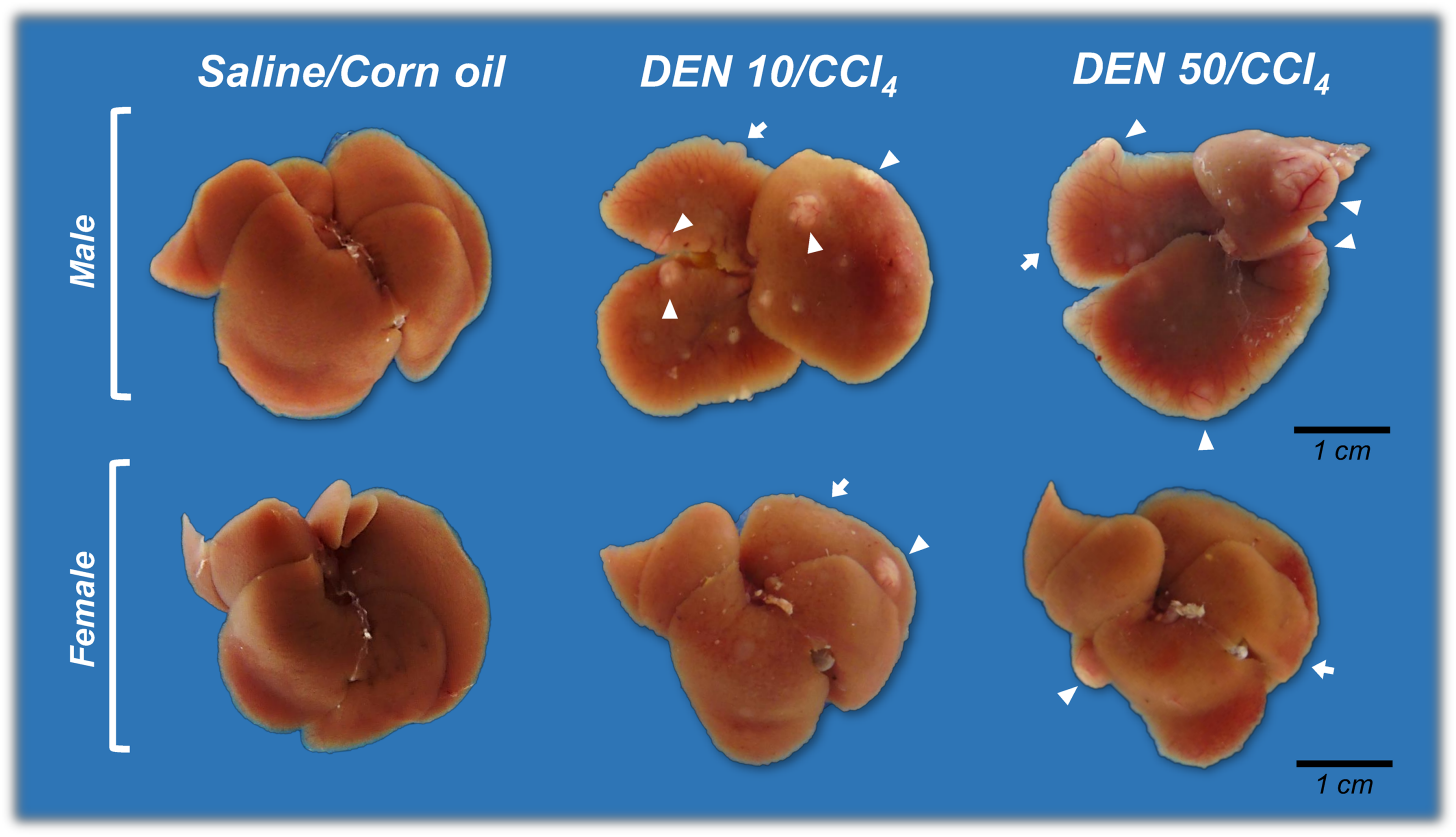
Representative macroscopic liver appearance of Saline/Corn oil and DEN/CCl_4_-treated male and female C3H/HeJ mice. DEN/CCl_4_-treated animals displayed typical “rough” fibrotic appearance (arrows) and developed whitish nodules (arrowheads). DEN 10 or 50 = diethylnitrosamine 10 or 50 mg/kg b.wt. in 0.9% saline at week 2, respectively and CCl_4_ = carbon tetrachloride, i.p., 0.25 to 1.50 μL/g b.wt. in 10% corn oil solution for 8 weeks (see Material and methods section).

At necropsy, in keeping with transaminases data, liver of male and female mice exposed to both DEN/CCl_4_ protocols showed irregular macroscopic surfaces, typical of liver fibrosis, while the liver of vehicle-treated mice showed normal smooth macroscopic appearance (Fig 2). Besides the fibrotic aspect, all DEN/CCl_4_-treated male mice developed multiple whitish macroscopic nodules (>1 mm in diameter) (p<0.001, for both), while vehicle-treated male mice did not (Fig 2, Table 2). Males from DEN 10/CCl_4_ and DEN 50/CCl_4_ groups demonstrated similar multiplicity of these gross alterations (5.87 ± 2.85 *vs*. 4.50 ± 2.20 nodules/mice, respectively, values are mean ± S.D.). The majority of females submitted to DEN 10/CCl_4_ (p<0.001) and DEN 50/CCl_4_ (p = 0.007) protocols developed these liver nodules as well (Fig 2, Table 2). The DEN 10/CCl_4_ and DEN 50/CCl_4_-treated females also showed similar multiplicity of these alterations [1.00 (1.00–1.50) *vs*. 1.00 (0.50–1.00) nodules/mice, respectively, values are median (q1-q3)]. All nodules were collected for further histopathological diagnosis.

**Table 2:** Effects of different DEN/CCl_4_ protocols on the incidence of macroscopic nodules, preneoplastic and neoplastic lesions of male and female C3H/HeJ mice.

Parameters	Groups^1^		
	Saline/Corn oil	DEN 10/CCl_4_	DEN 50/CCl_4_
*Males*			
Macroscopic nodules	0/8 (0)^b^	8/8 (100%)^a^	8/8 (100%)^a^
All types of AHF	0/8 (0)^b^	8/8 (100%)^a^	8/8 (100%)^a^
Basophilic AHF	-	8/8 (100%)	8/8 (100%)
Eosinophilic AHF	-	3/8 (37.5%)	6/8 (75%)
Clear cell AHF	-	8/8 (100%)	4/8 (50%)
HCA	0/8 (0)^b^	8/8 (100%)^a^	6/8 (75%)^a^
HCC	0/8 (0)	1/8 (12.5%)	0/8 (0)
*Females*			
Macroscopic nodules	0/8 (0)^b^	7/8 (87.5%)^a^	6/8 (75%)^a^
All types of AHF	0/8 (0)^b^	8/8 (100%)^a^	8/8 (100%)^a^
Basophilic AHF	-	7/8 (87.5%)	8/8 (100%)
Eosinophilic AHF	-	4/8 (50%)	6/8 (75%)
Clear cell AHF	-	2/8 (25%)	3/8 (37.5%)
HCA	0/8 (0)^b^	5/8 (62.5%)^a^	2/8 (25%)^ab^

Values are the proportion of affected mice (percentage).

^1^DEN 10 or 50 = diethylnitrosamine 10 or 50 mg/kg b.wt. in 0.9% saline at week 2 and CCl_4_ = carbon tetrachloride, i.p., 0.25 to 1.50 μL/g b.wt. in 10% corn oil solution for 8 weeks (see Material and methods section).

AHF = altered hepatocyte foci; HCA = hepatocellular adenoma; HCC = hepatocellular carcinoma. Different letters correspond to statistical difference among groups by Fisher’s exact test (p<0.05).

Finally, considering the fibrotic aspect and the presence of nodules, male mice submitted to DEN 10/CCl_4_ protocol displayed enhanced liver absolute and relative weights (p<0.001, for both) compared to vehicle group, whereas DEN 50/CCl_4_-protocol just enhanced liver relative weight (p<0.001) (Table 1). In females, all DEN/CCl_4_ protocols increased liver relative and absolute weights compared to vehicle-treated group (p<0.001, for both) (Table 1).

### Histopathological evaluation

**Fig 3:**
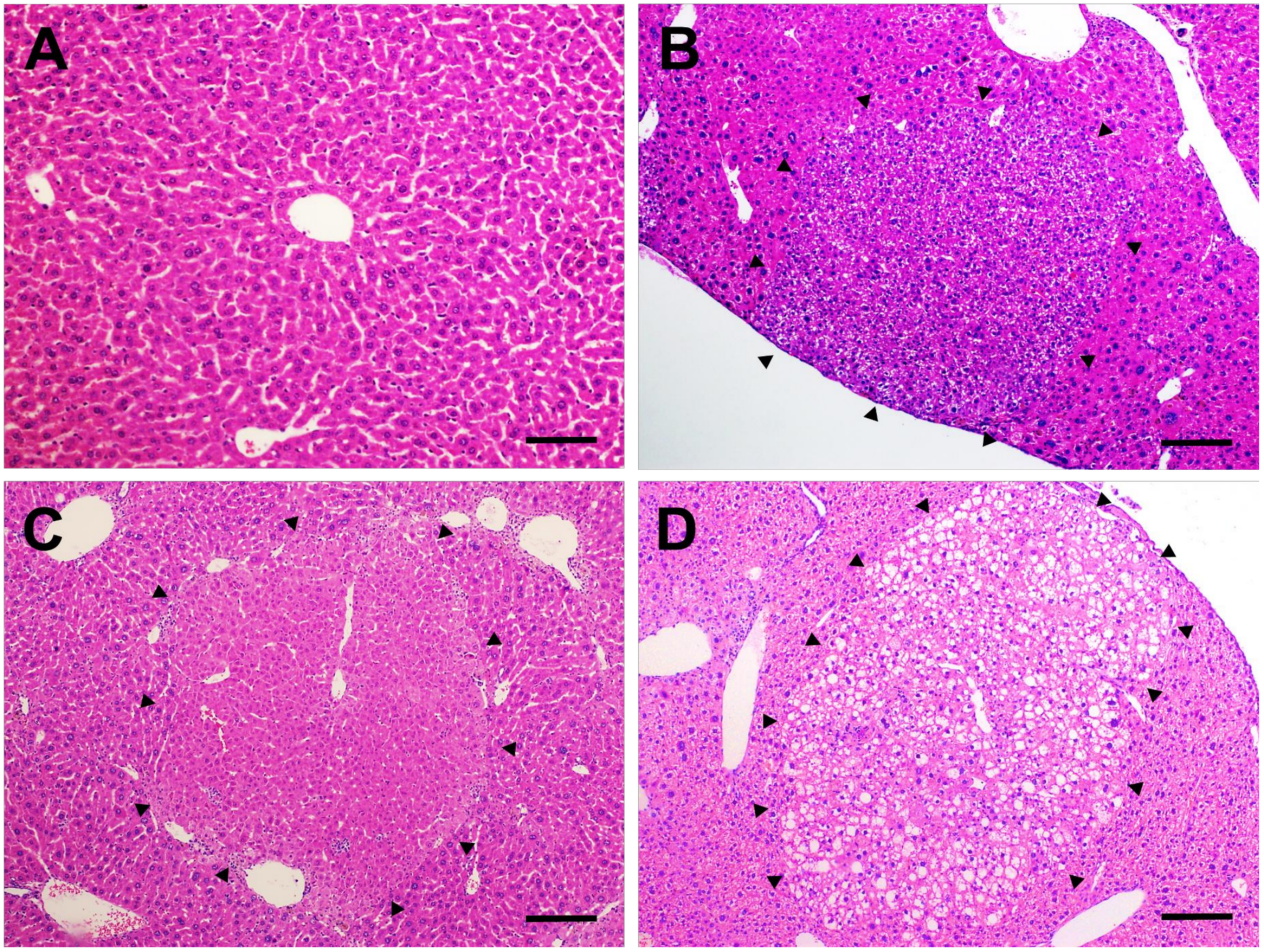
Preneoplastic altered hepatocyte foci. Representative photomicrographs of **(A)** normal liver of vehicle-treated groups (20× objective) (scale bar = 50 μm) and **(B)** basophilic, **(C)** eosinophilic and **(D)** clear cell preneoplastic foci found in the liver of DEN/CCl_4_-treated mice (10× objective) (scale bar = 100 μm).

**Fig 4:**
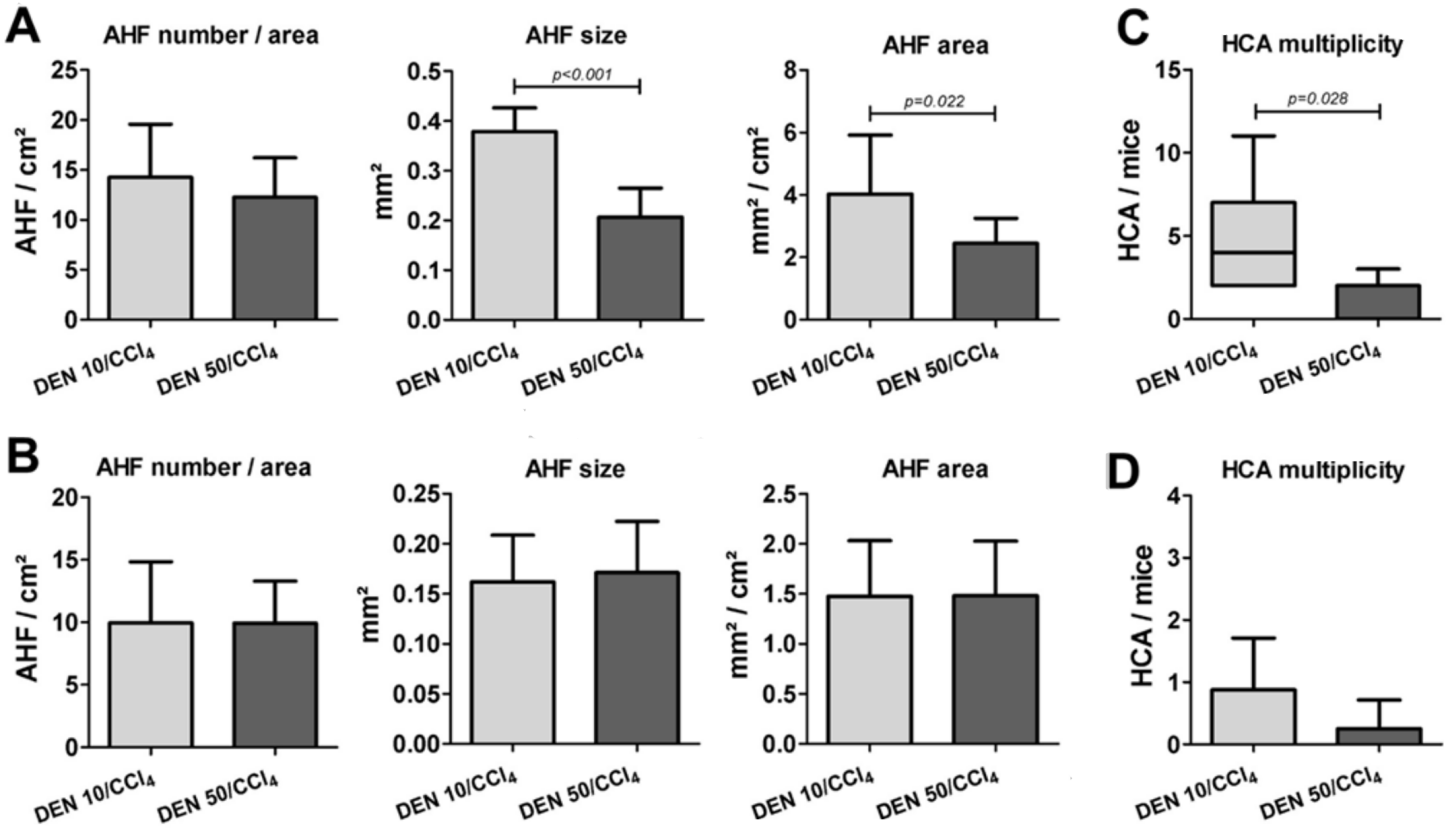
Effects of different DEN/CCl_4_ protocols on AHF analysis (number/area, size and area) and HCA multiplicity of male and female C3H/HeJ mice. (A, C) male and (B, D) female. Values are mean + S.D or box and whiskers. n = 8 mice/sex/group. DEN 10 or 50 = diethylnitrosamine 10 or 50 mg/kg b.wt. in 0.9% saline at week 2, respectively and CCl_4_ = carbon tetrachloride, i.p., 0.25 to 1.50 μL/g b.wt. in 10% corn oil solution for 8 weeks (see Material and methods section). AHF = altered hepatocyte foci; HCA = hepatocellular adenoma. Data were analyzed by Student t or Mann-Whitney test (p<0.05).

The histopathological evaluation of liver samples from males and females revealed the occurrence of preneoplastic AHF, sub-classified as basophilic, eosinophilic or clear cell foci, according to the predominant cell type (Fig 3). In both DEN/CCl_4_ regimens applied in males and females, all mice developed AHF, and these groups presented enhanced incidence of these preneoplastic lesions (considering all types) when compared to corresponding vehicle controls (p<0.001, for all) (Table 2). Considering the different types of AHF, DEN 10/CCl_4_ and DEN 50/CCl_4_ protocols induced similar incidence of basophilic, eosinophilic and clear cell foci in both males and females (Table 2). In our models, at the chosen time-point (week 17), the occurrence of basophilic AHF displayed increased importance, since all males and the majority of females developed these specific lesions (Table 2). Also, male mice submitted to DEN 10/CCl_4_ and DEN 50/CCl_4_ protocols displayed similar number of AHF *per* liver area (cm^2^) (Fig 4). Interestingly, DEN 10/CCl_4_ protocol displayed an increase in mean AHF size (p<0.001) and total liver area occupied by all these lesions (p = 0.022) compared to DEN 50/CCl_4_ group (Fig 4). In our female models, the all protocols did not differ in all parameters of AHF analysis (Fig 4).

In addition to preneoplastic lesions, the histopathological evaluation of liver nodules revealed the presence of neoplastic lesions, represented by HCA and HCC (S2 Fig). In male mice, we observed a significant increase in HCA incidence in both DEN 10/CCl_4_ (p<0.001) and DEN 50/CCl_4_ (p = 0.007) protocols compared to vehicle-treated mice (Table 2). Yet similar in incidence (Table 2), the DEN 10/CCl_4_ protocol resulted in an increase in HCA multiplicity in comparison to DEN 50/CCl_4_-treated group (p = 0.028) (Fig 4). Lastly, although 12.5% of males in DEN 10/CCl_4_ group developed HCC, our male models did not increase the incidence of this malignant lesion compared to vehicle group (Table 2). At the chosen time-point, female mice did not develop HCCs, only HCAs. However, only DEN 10/CCl_4_ females significantly enhanced HCA incidence compared to vehicle-treated mice (p = 0.026) (Table 2). No significant changes were observed between DEN/CCl_4_ female protocols in relation to HCA multiplicity (Fig 4).

Interestingly, the DEN 10/CCl_4_ protocol in males and females displayed better results than DEN 50/CCl_4_ protocol on inducing the development of AHF and/or HCA. Especially in DEN 10/CCl_4_ models, the data on incidence and multiplicity indicate that these hepatocellular lesions can be applied as reliable end-point lesions.

### Proliferation and apoptosis analysis

**Fig 5:**
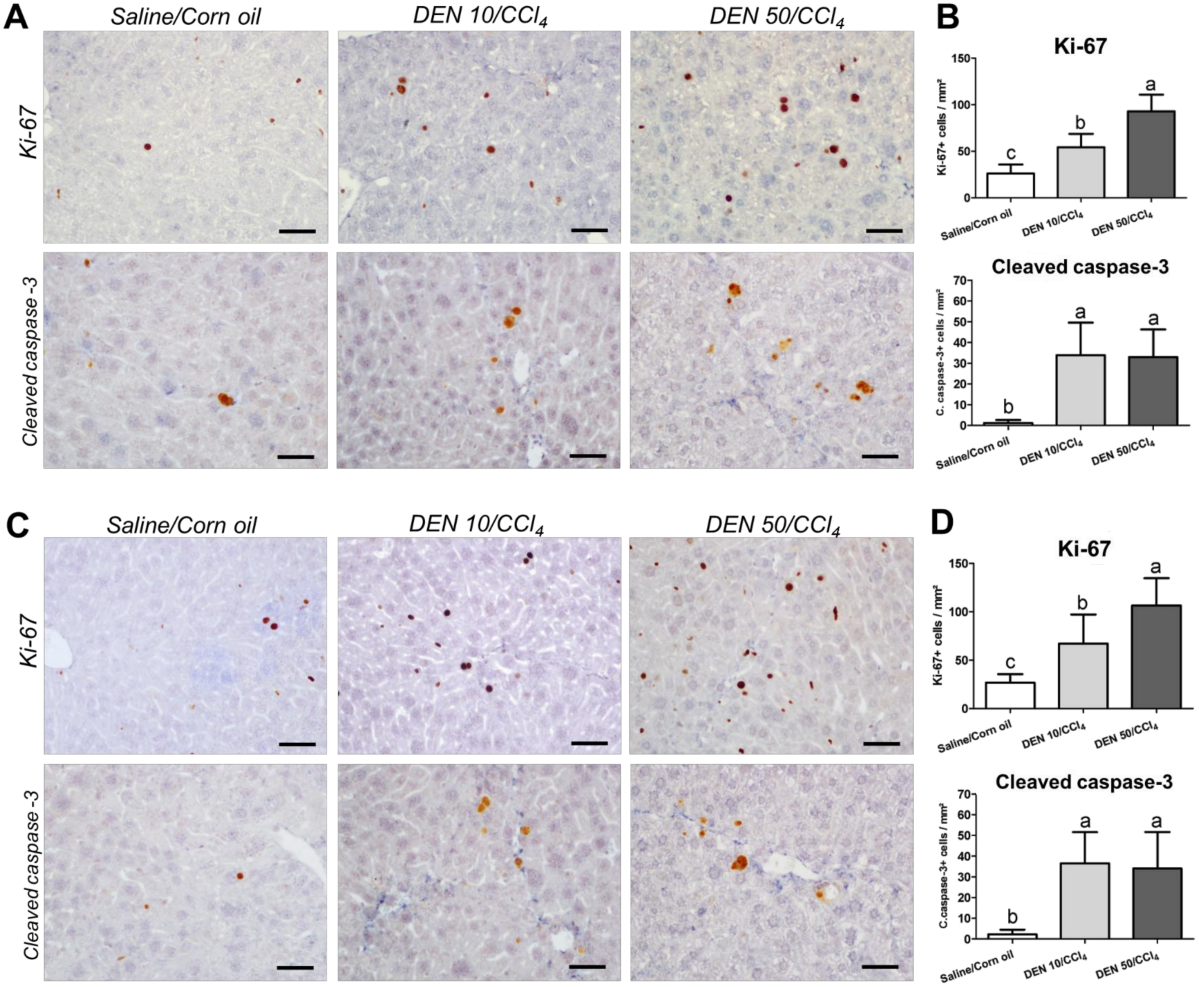
Effects of different DEN/CCl_4_ protocols on Ki-67 (cell proliferation) and cleaved caspase-3 (apoptosis) immunostaining in surrounding liver tissue of male and female C3H/HeJ mice. (A, B) male and (C, D) female. (A, C) Representative photomicrographs (40× objective) (scale bar = 20 μm) and (B, D) semiquantitative analysis. Values are mean + S.D. n = 8 mice/sex/group. DEN 10 or 50 = diethylnitrosamine 10 or 50 mg/kg b.wt. in 0.9% saline at week 2, respectively and CCl_4_ = carbon tetrachloride, i.p., 0.25 to 1.50 μL/g b.wt. in 10% corn oil solution for 8 weeks (see Material and methods section). Different letters correspond to statistical difference among groups by ANOVA and *post hoc* Tukey’s test (p<0.05).

Sustained cell proliferation could favor the clonal expansion of DEN-initiated hepatocytes and predispose mice to the development of hepatocellular preneoplastic and neoplastic [23]. Furthermore, increased apoptosis after liver injury may also contribute to the initiation phase of HSCs activation and ultimately, to liver fibrosis [24]. In the proposed models, when analyzed the “normal-appearing” liver tissue (avoiding preneoplastic and neoplastic lesions), both DEN/CCl_4_ protocols significantly increased hepatocyte proliferation (Ki-67) and apoptosis (cleaved caspase-3) labeling indexes in the liver from males (p<0.001, for both) and females (p = 0.002 and p = 0.0012, respectively) in comparison to corresponding vehicle-treated controls (Fig 5). DEN 50/CCl_4_ regimens in males and females resulted in an increased hepatocyte proliferation in comparison DEN10/CCl_4_ (Fig 5). No significant changes were observed between DEN/CCl_4_ protocols in relation to hepatocyte apoptosis indexes (Fig 5).

**Fig 6:**
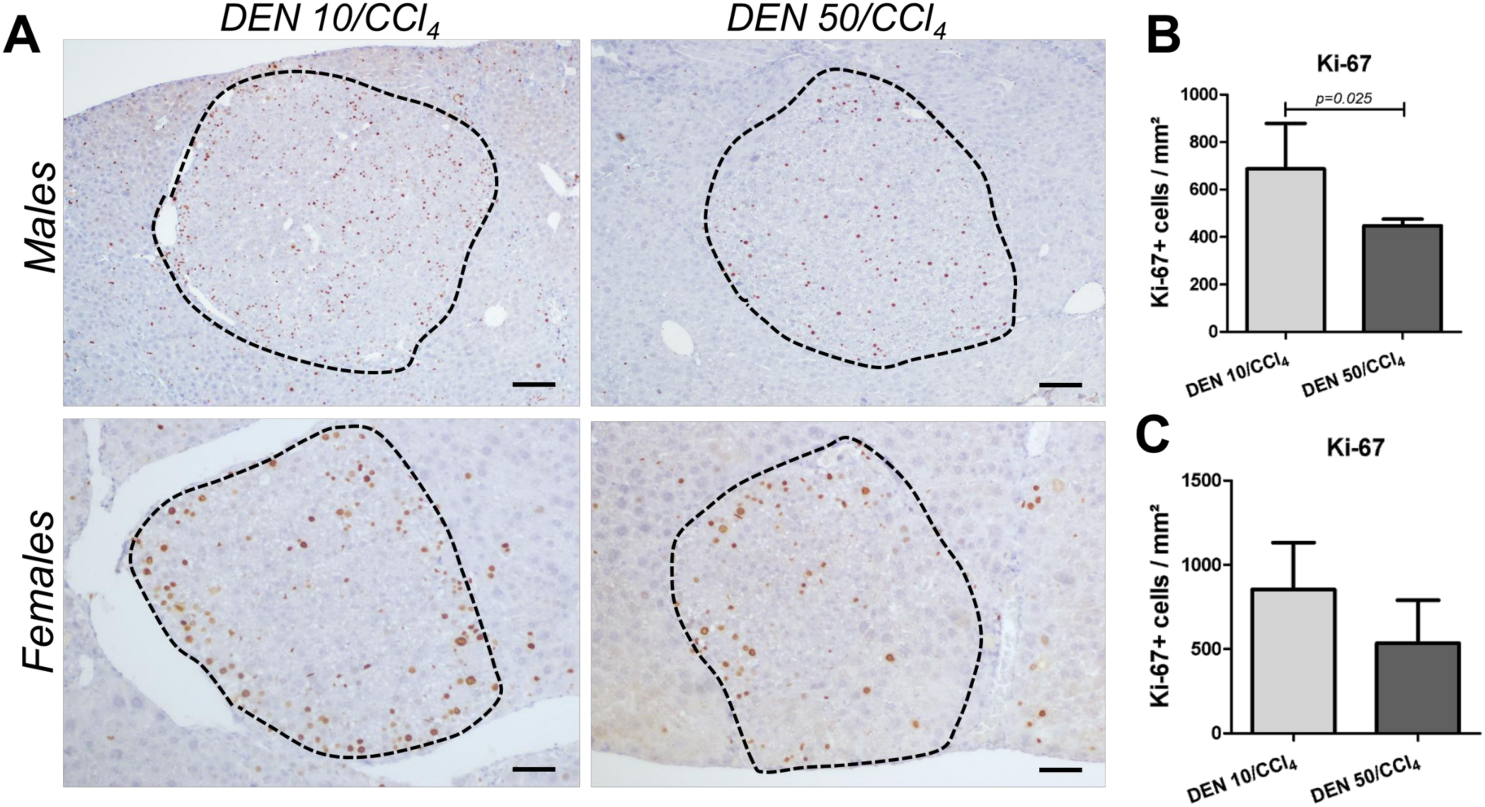
Effects of different DEN/CCl_4_ protocols on Ki-67 (cell proliferation) immunostaining in preneoplastic AHF of male and female C3H/HeJ mice. (A, B) male and (A, C) female. (A) Representative photomicrographs (Males: 10× objective, scale bar = 100 μm) (Females: 20× objective, scale bar = 50 μm) and (B, D) semiquantitative analysis. Values are mean + S.D. n = 8 mice/sex/group. DEN 10 or 50 = diethylnitrosamine 10 or 50 mg/kg b.wt. in 0.9% saline at week 2, respectively and CCl_4_ = carbon tetrachloride, i.p., 0.25 to 1.50 μL/g b.wt. in 10% corn oil solution for 8 weeks (see Material and methods section). AHF = altered hepatocyte foci. Data were analyzed by Student t test (p<0.05).

Despite of presenting increased cell proliferation in “surrounding” liver tissue, DEN 50/CCl_4_ regimen in males showed diminished hepatocyte proliferation into AHF in comparison to DEN 10/CCl_4_ (p = 0.025) (Fig 6). This data corroborates with increased AHF size and area displayed by DEN 10/CCl_4_ protocol (Fig 4). Hepatocyte proliferation into preneoplastic AHF favors the accumulation of molecular alterations that predispose these lesions to growth and progression to neoplastic lesions [23]. In females, no significant changes were observed between DEN/CCl_4_ protocols in relation to cell proliferation into AHF (Fig 6A and 6C), in accordance to the AHF analysis in this sex (Fig 4).

DEN 10/CCl_4_ and 50/CCl_4_ male protocols were similar in HCA proliferation rates (520.36 ± 121.80 vs. 497.75 ± 55.68 Ki-67^+^ cells/mm^2^, respectively. Values are mean ± S.D.). Lastly, we did not characterize the cell proliferation rates in female HCA considering the non-significant incidence of these lesions in DEN 50/CCl_4_ females (Table 2). Representative photomicrographs of Ki-67 immunostained sections of HCA and HCC are presented in S2 Fig.

### β-catenin and TGF-α evaluation

The aberrant activation of Wnt/β-catenin signaling is frequently observed in human and mouse hepatocarcinogenesis [25], conferring to the hepatocytes the capacity of sustained cell proliferation. Nonetheless, all animals of both female and male models presented typical membranous expression of β-catenin in surrounding liver tissue, altered hepatocyte foci (all types) and hepatocellular adenomas. As expected, western blot revealed similar hepatic β-catenin protein expression in both male and female models compared to their respective controls (S3 Fig). Consistently to our findings, a previous DEN/CCl_4_-induced model in B6C3F1 mice also led to typical membranous β-catenin staining and did not display codon 2 mutations in *Ctnnb1* gene [26]. Indeed, at the analyzed time-point, cytoplasmic/nuclear and increased β-catenin expression did not show to be marked feature of our models.

The transforming growth factor-α (TGF-α), which binds to the epidermal growth factor receptor (EGFR), also act as a potent mitogenic signal to hepatocytes and it has been implicated in rat, mice and human hepatocarcinogenesis [17, 27, 28]. In all groups of male and female models, mice showed typical staining on endothelial and bile duct cells (S4 Fig) and only few DEN 10/CCl_4_ [female: 25% (2/8); male: 12.5% (1/8)] and DEN 50/CCl_4_ [female: 12.5% (1/8); male: 25% (2/8)] mice developed weakly stained TGF-α-positive foci (S4 Fig). Interestingly, in keeping with previous report [27], all TGF-α positive foci in these few animals displayed eosinophilic phenotype (S4 Fig). Basophilic, clear cell foci and adenomas (S4 Fig) did not present TGF-α immunoreactivity. Despite of being a marker of progression in DEN-initiated and DEN-initiated/PB-promoted mice models, [27], the TGF-α pathway was not significantly activated in our DEN-initiated/CCl_4_-promoted bioassays after 17 weeks of experiment.

### Fibrosis analysis: Collagen, α-SMA and CD68

**Fig 7:**
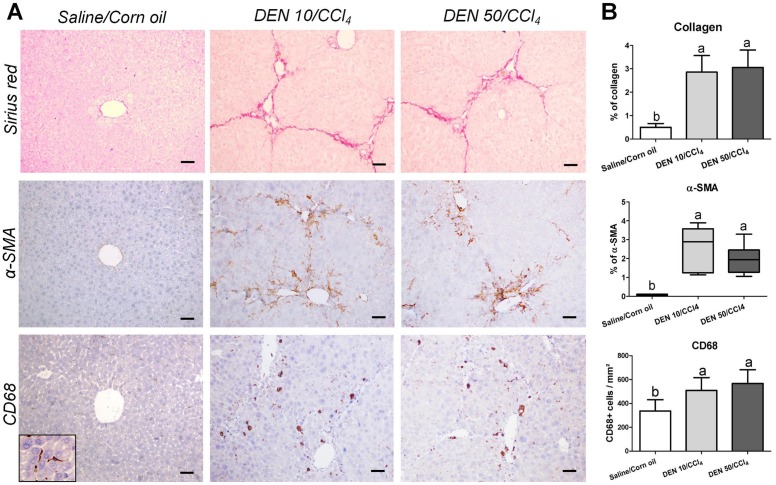
Effects of different DEN/CCl_4_ protocols on collagen content (Sirius red), α-SMA and CD68 immunostaining in the liver of male C3H/HeJ mice. (A) Representative photomicrographs (20× objective; scale bar = 50 μm) and (B) semiquantitative analysis. Values are mean + S.D or box and whiskers. n = 8 mice/group. DEN 10 or 50 = diethylnitrosamine 10 or 50 mg/kg b.wt. in 0.9% saline at week 2, respectively and CCl_4_ = carbon tetrachloride, i.p., 0.25 to 1.50 μL/g b.wt. in 10% corn oil solution for 8 weeks (see Material and methods section). Different letters correspond to statistical difference by ANOVA or Kruskal Wallis and *post hoc* Tukey test or Dunn’s method, respectively (p<0.05).

In keeping with the enhanced transaminases levels (Table 1) and irregular macroscopic appearance (Fig 2), Sirius red-stained liver sections of males from DEN/CCl_4_-treated protocols revealed fibrous expansions in portal areas and marked bridging between portal areas and central veins, typical features of liver fibrosis (Fig 7). As expected, morphometric analysis demonstrated increased collagen deposition in the liver from DEN/CCl_4_-treated groups compared to vehicle-treated group (p<0.001) (Fig 7). Moreover, these groups displayed marked activation of HSCs, considering the increased α-SMA immunostaining (p = 0.0036), in the same areas of fibrous expansion and bridging (Fig 7). In addition to HSCs, CD68^+^ liver macrophages already showed to play important roles on inducing pro-inflammatory and pro-fibrogenic responses in mice and humans [28]. Here, upon both DEN/CCl_4_ regimens, we found a significant increase in the number of CD68^+^ cells in the liver (p = 0.0057). In fibrotic liver, these cells were concentrated in areas surrounding the fibrous expansions, while in untreated mice, CD68^+^ macrophages were detected in the sinusoids (detail in Fig 7).

**Fig 8:**
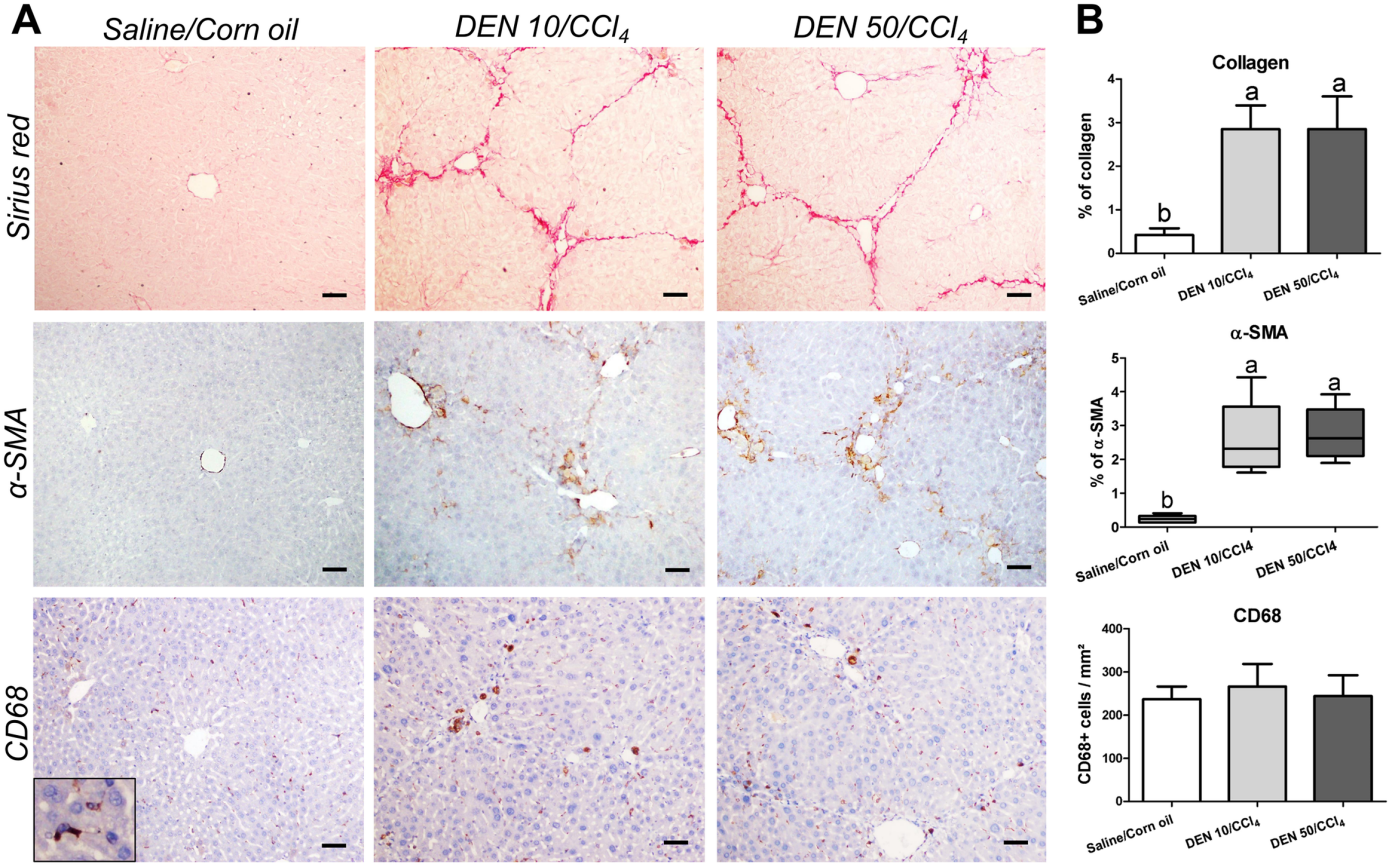
Effects of different DEN/CCl_4_ protocols on collagen content (Sirius red), α-SMA and CD68 immunostaining in the liver of female C3H/HeJ mice. (A) Representative photomicrographs (20× objective; scale bar = 50 μm) and (B) semiquantitative analysis. Values are mean + S.D or box and whiskers. n = 8 mice/group. DEN 10 or 50 = diethylnitrosamine 10 or 50 mg/kg b.wt. in 0.9% saline at week 2, respectively and CCl_4_ = carbon tetrachloride, i.p., 0.25 to 1.50 μL/g b.wt. in 10% corn oil solution for 8 weeks (see Material and methods section). Different letters correspond to statistical difference by ANOVA or Kruskal Wallis and *post hoc* Tukey test or Dunn’s method, respectively (p<0.05).

In females, fibrotic response to CCl_4_ protocol was similar to males. The DEN 10/CCl_4_ and DEN 50/CCl_4_ groups also showed clear features of liver fibrosis, increased collagen deposition (p<0.0001) and enhanced α-SMA immunostaining (p = 0.0087) (Fig 8). Although CD68^+^ macrophages were also mainly found near the scar tissue, the number of these cells *per* mm^2^ remained unchanged in fibrotic liver (Fig 8). Data indicate that the CCl_4_ administrations were successful on establishing a similar fibrotic background in male and female C3H/HeJ mice. In addition, the different protocols of DEN-induced hepatocarcinogenesis (10 and 50 mg/Kg b.wt.) did not influence the collagen deposition and HSCs activation at the end of experimental period.

### NF-κB, COX-2 and IL-6 analysis

Both IkB kinase β (IKKβ), part of NF-κB transcription factor, and cyclooxygenase-2 (COX-2), an enzyme that synthesizes prostaglandins, are related to the up regulation of interleukin-6 (IL-6) [29, 30]. Besides its role in acute inflammation, this multifunctional cytokine considered is a strong hepatomitogen involved in experimental liver carcinogenesis [7]. Indeed, in a DEN-induced neonatal mice model, IL-6 knockout attenuated hepatocarcinogenesis, reassuring the importance of IL-6 axis in this process even in the absence of liver fibrosis [7].

**Fig 9:**
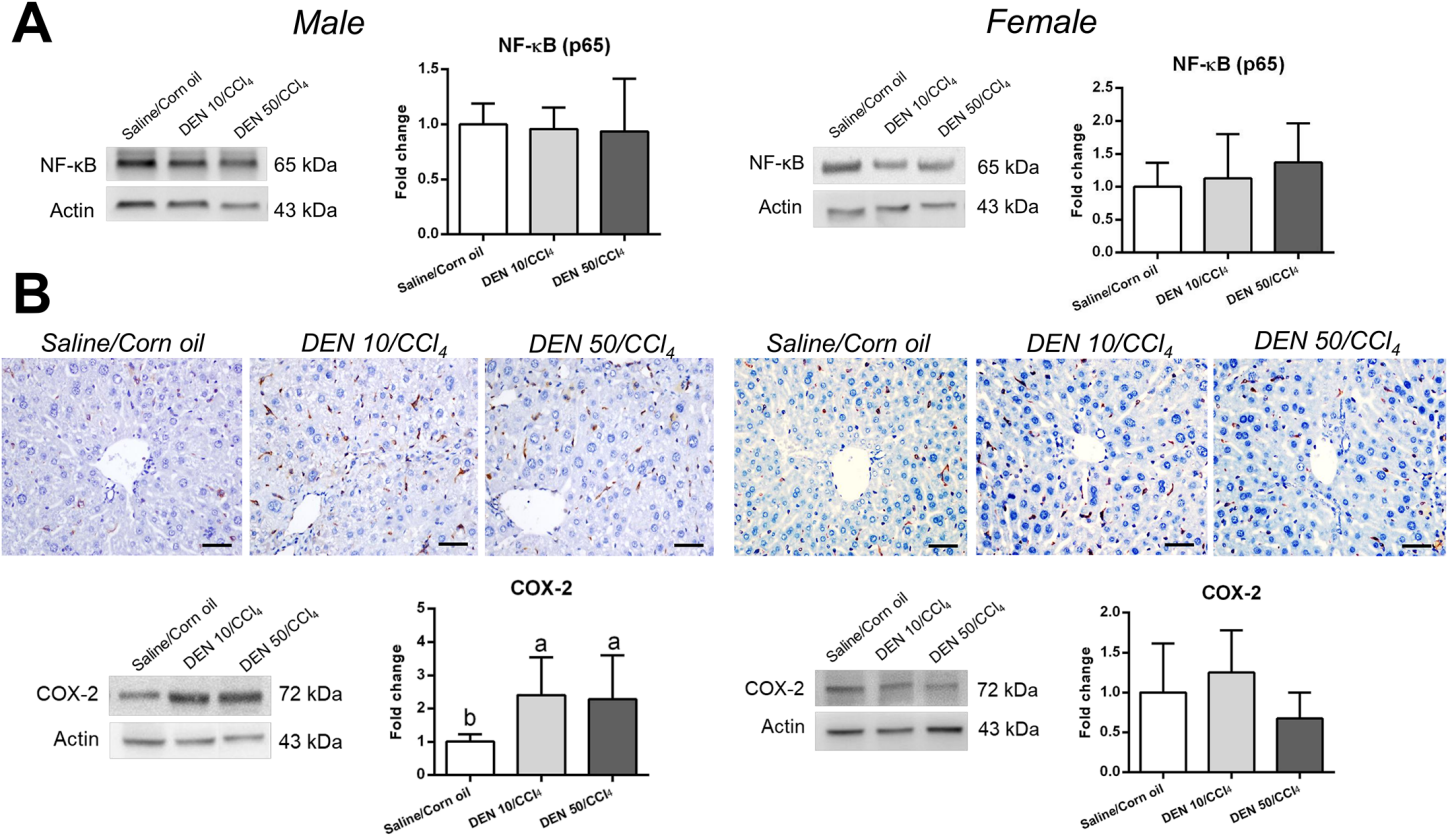
Effects of different DEN/CCl_4_ protocols on (A) NF-κB (p65) protein expression and (B) COX-2 protein immunostaining and expression in the liver of male and female C3H/HeJ mice. Representative photomicrographs (40× objective; scale bar = 20 μm), western blot bands and semiquantitative analysis are presented. Values are mean + S.D. n = 6 mice/group. DEN 10 or 50 = diethylnitrosamine 10 or 50 mg/kg b.wt. in 0.9% saline at week 2, respectively and CCl_4_ = carbon tetrachloride, i.p., 0.25 to 1.50 μL/g b.wt. in 10% corn oil solution for 8 weeks (see Material and methods section). Different letters correspond to statistical difference among groups by ANOVA and *post hoc* Tukey’s test (p<0.05).

**Fig 10:**
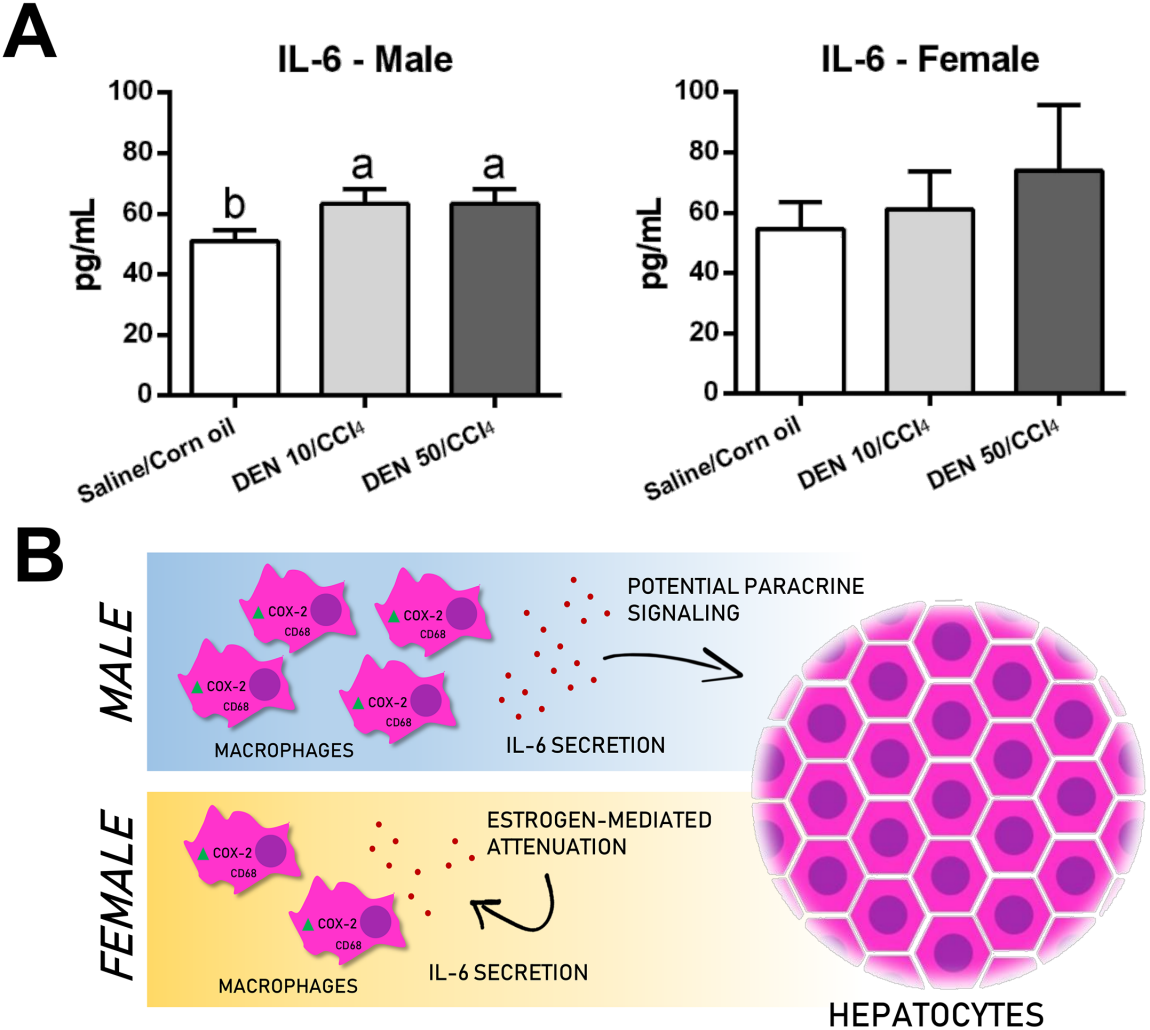
(A) Effects of different DEN/CCl_4_ protocols on interleukin 6 (IL-6) levels in the liver of male and female C3H/HeJ mice. (B) Sex differences on CD68/COX-2/IL-6 axis in the proposed models. Values are mean + S.D. n = 6 mice/group. DEN 10 or 50 = diethylnitrosamine 10 or 50 mg/kg b.wt. in 0.9% saline at week 2, respectively and CCl_4_ = carbon tetrachloride, i.p., 0.25 to 1.50 μL/g b.wt. in 10% corn oil solution for 8 weeks (see Material and methods section). Different letters correspond to statistical difference among groups by ANOVA and *post hoc* Tukey’s test (p<0.05).

Despite of not altering p65 protein expression, both male models significantly enhanced COX-2 protein expression and IL-6 levels in the liver compared to their untreated counterpart (p = 0.0092 and p<0.0001, respectively) (Figs 9 and 10). These groups displayed many COX-2^+^ mononuclear inflammatory cells in the liver while untreated group, few COX-2^+^ in cells were found in the sinuosoids (Fig 9). In chronic or acute thioacetamide- or CCl_4_ induced liver damage, COX-2 mRNA expression is increased and restricted to mononuclear phagocytes [31]. In human cirrhosis, COX-2^+^ cells showed to be CD68^+^ as well [31]. Thus, the enhanced number of CD68^+^ macrophages in our male models corroborates with the increase in COX-2/IL-6 axis. Macrophages are responsible for IL-6 secretion and potential paracrine signaling on neighboring hepatocytes, contributing to preneoplastic and neoplastic lesion development [7]. In males, CD68/COX-2/IL-6 axis emerges as a molecular target for potential preventive or therapeutic strategies.

In contrast, female models showed no differences on IL-6 levels, COX-2 and NF-κB protein expression and displayed similar immunolocalization of COX-2^+^ cells compared to Saline/Corn oil group (Figs 9 and 10). These data also endorse CD68 findings in female models. Although we characterized standard male and female models separately, we performed a sex comparison in order to confirm sex bias. Our models successfully recapitulated this feature of hepatocarcinogenesis, since male models displayed enhanced serum ALT levels [DEN 10/CCl_4_ (p = 0.020); DEN 50/CCl_4_ (p = 0.001)], preneoplastic AHF with increased size (only in DEN 10/CCl_4_ model, p<0.001), area (p = 0.008, for both models) and enhanced HCA multiplicity [DEN 10/CCl_4_ (p = 0.007); DEN 50/CCl_4_ (p = 0.020)] compared to females (S5 Fig). Our data suggests clear sex difference, supporting previous studies showing that estrogen mediates a reduction in IL-6 signaling, leading to decreased hepatocarcinogenesis susceptibility in females [7].

### Lipid peroxidation and antioxidant defense

**Fig 11:**
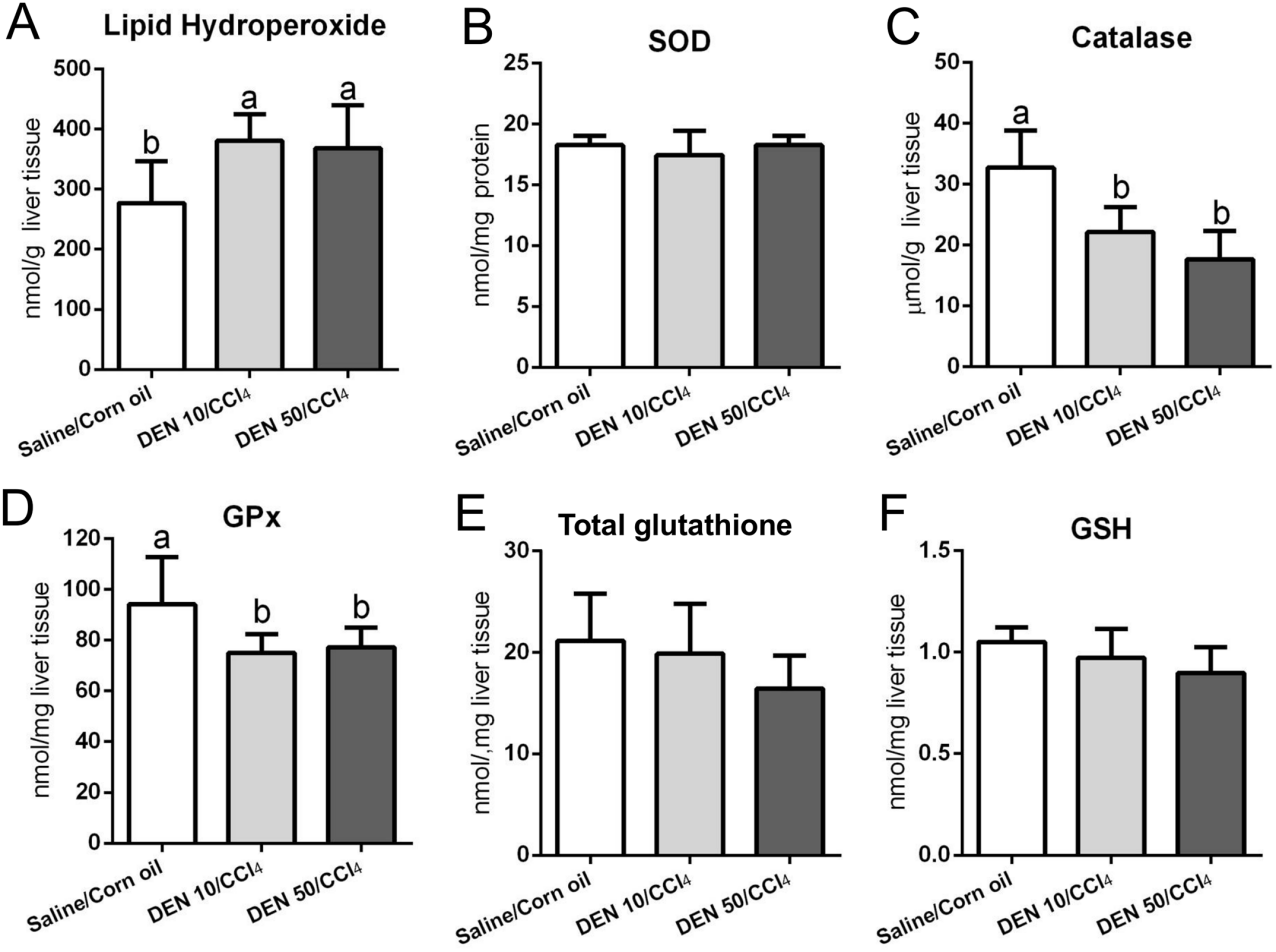
Effects of different DEN/CCl_4_ protocols on (A) lipid hydroperoxide levels and (B-F) antioxidant defense in the liver of male C3H/HeJ mice. Values are mean + S.D. n = 8 mice/group. SOD = superoxide dismutase; GSH-Px = glutathione peroxidase; GSH = reduced glutathione; DEN 10 or 50 = diethylnitrosamine 10 or 50 mg/kg b.wt. in 0.9% saline at week 2, respectively and CCl_4_ = carbon tetrachloride, i.p., 0.25 to 1.50 μL/g b.wt. in 10% corn oil solution for 8 weeks (see Material and methods section). Different letters correspond to statistical difference among groups by ANOVA and *post hoc* Tukey’s test (p<0.05).

**Fig 12:**
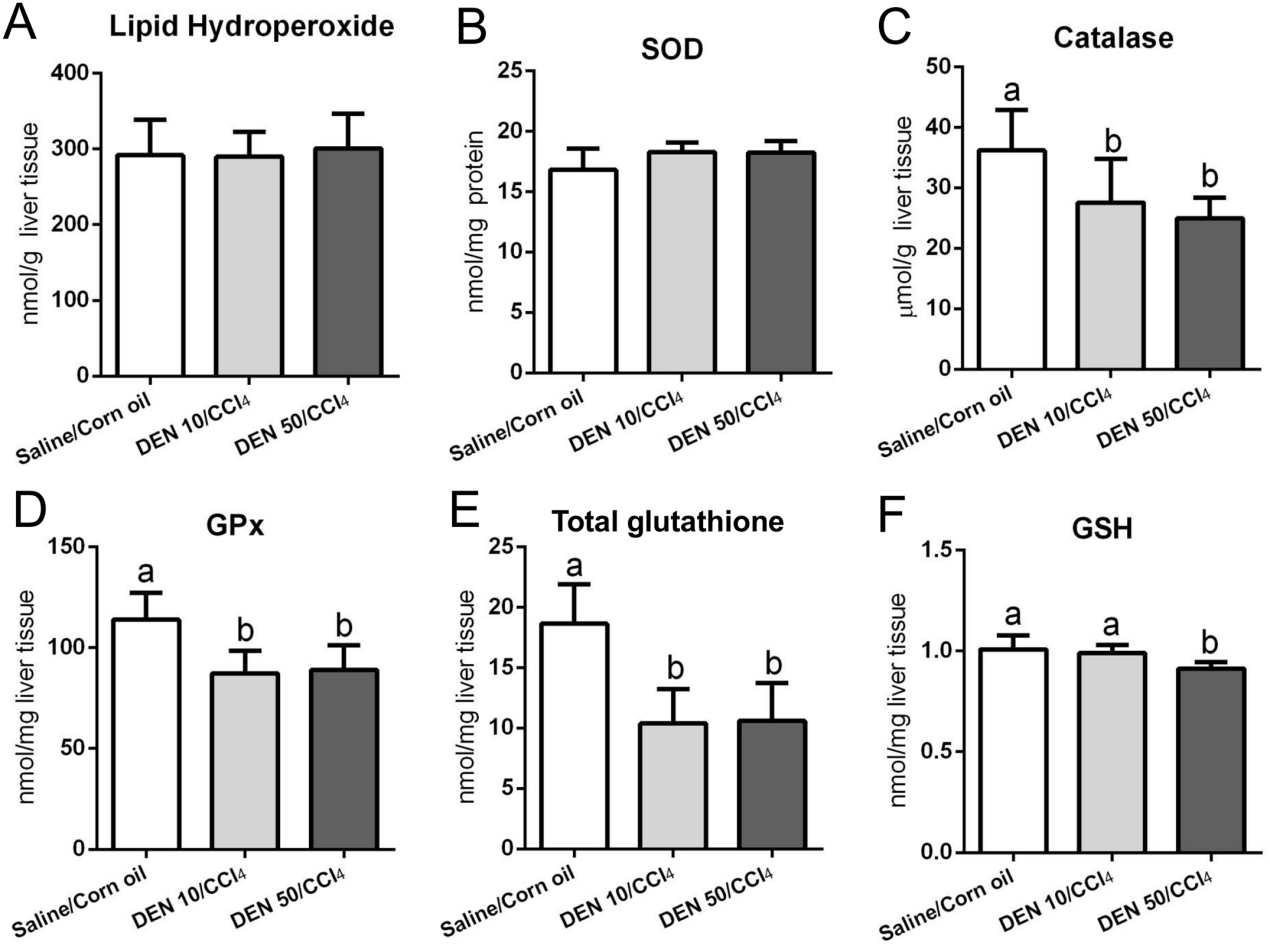
Effects of different DEN/CCl_4_ protocols on (A) lipid hydroperoxide levels and (B-F) antioxidant defense in the liver of female C3H/HeJ mice. Values are mean + S.D. n = 8 mice/group. SOD = superoxide dismutase; GSH-Px = glutathione peroxidase; GSH = reduced glutathione; DEN 10 or 50 = diethylnitrosamine 10 or 50 mg/kg b.wt. in 0.9% saline at week 2, respectively and CCl_4_ = carbon tetrachloride, i.p., 0.25 to 1.50 μL/g b.wt. in 10% corn oil solution for 8 weeks (see Material and methods section). Different letters correspond to statistical difference among groups by ANOVA and *post hoc* Tukey’s test (p<0.05).

The hepatic metabolism of CCl_4_ results in trichloromethyl radicals, which are involved in lipid peroxidation and contribute to chronic liver damage and fibrosis [32,33]. Indeed, both protocols applied in male mice increased lipid hydroperoxide levels compared to vehicle-treated group (p = 0.013) (Fig 11) while those applied in females did not (Fig 12). Our male models also showed diminished activities of catalase and GSH-Px enzymes (p<0.001 and p = 0.014, respectively) and no effect on SOD, GSH and total glutathione (Fig 11), a week after the last CCl_4_ administration. Females submitted to both DEN/CCl_4_ regimens demonstrated decreased activity of catalase (p = 0.003), GSH-Px (p<0.001) and reduced levels of total glutathione (p<0.001) compared to the vehicle group (Fig 12). The DEN 50/CCl_4_ protocol reduced GSH levels as well (p = 0.002) (Fig 12), but no difference was observed on SOD levels in both groups (Fig 12).

The catalase enzyme and the glutathione system (mainly GSH-Px and its non-enzymatic GSH substrate) can directly avoid the oxidative degradation of lipids by neutralizing reactive radicals [34]. Thus, the depletion or diminished activity of these antioxidant agents is a consequence of CCl_4_ chronic administration. An impairment in antioxidant axis is a marked feature of both human and rodent hepatocarcinogenesis [6,12,35,36], predisposing mice to the development of preneoplastic and neoplastic lesions.

## Discussion

In the current study, we proposed an early fibrosis-associated hepatocarcinogenesis model for male and female mice, focusing on establishing standard mice models and minimizing the required experimental time for the development of hepatocellular preneoplastic and neoplastic lesions in a fibrotic background. For this purpose, we applied DEN/CCl_4_ regimen in a hepatocarcinogenesis-susceptible C3H/HeJ mice strain. After DEN administration in neonatal mice, this carcinogen is metabolized in the liver by CYP2E1, generating reactive oxygen species (ROS) and the nucleophilic ethyldiazonium ions, which lead to DNA damage and genomic instability, “initiating” hepatocytes for carcinogenesis [37,38]. In neonatal age, increased cell proliferation rates in the developing liver facilitates the clonal expansion of DEN-initiated hepatocytes [10]. The main outcome of this feature is earlier occurrence of preneoplastic and neoplastic lesions compared to classical models that DEN initiation occurs in fully-developed liver of adult mice [39]. Besides, in order to promote DEN-induced liver lesions, we established a fibrotic microenvironment by administrating successive CCl_4_ injections to mimic the chronic liver injury as in human liver disease. The CYP2E1 is also responsible for CCl_4_ biotransformation, which leads to the formation of trichloromethyl radical, involved in free radical and lipid peroxidation reactions [32,33]. The chronic CCl_4_ administration contributes to the necrosis of centrilobular hepatocytes and the induction of a chronic inflammatory response, which promote HSCs activation and hence collagen deposition [40].

At the chosen time-point (week 17), we observed a clear liver fibrosis in both DEN doses in male mice (DEN10/CCl_4_ and DEN50/CCl_4_), characterized by increased collagen accumulation (Sirius red), HSCs activation (α-SMA), increased population of CD68^+^ macrophages and activation of COX-2/IL-6 axis. In addition, liver of males displayed increased oxidative stress (lipid hydroperoxide) and decreased activity of antioxidant enzymes (catalase and GSH-Px). Moreover, the two protocols resulted in increased cell proliferation (Ki-67) and apoptosis (cleaved caspase-3) in liver tissue surrounding the hepatocellular preneoplasic/neoplasic lesions. Regarding these lesions, all DEN10/CCl_4_ and DEN50/CCl_4_-treated male mice developed preneoplastic AHFs and HCAs whereas the vehicle group did not. Previous findings indicate that 10% to 15% of the DEN-induced AHFs in C3H/HeJ and B6C3F1 mice strains presented mutations in proliferation-related *H-ras* gene [41,42]. Indeed, the enhanced expression of mutant *H-ras* alone is sufficient to induce hepatocyte proliferation and AHF growth [43]. Under growth stimuli, AHF can eventually accumulate other genetic and epigenetic alterations and progress from focal lesions to neoplastic lesions, as HCAs and HCCs [23]. For these reasons, AHF are considered putative hepatocellular preneoplastic lesions in mice [23]. Although HCAs are benign lesions and their progression to HCCs is not well documented in mice, HCAs display progressive atypia and ~50% also exhibit *H-ras* mutations in C3H/HeJ mice [42,44]. Besides, a recent study showed that both HCA and HCC cells in DEN/CCl_4_-induced mice neoplasms are derived from the same genetic cell lineage [45], suggesting that HCAs can be precursor lesions for HCC. Therefore, in medium-term chemically-induced hepatocarcinogenesis bioassays proposed, both AHFs and HCAs can be considered suitable end-point lesions, permitting the screening of potential modifying factors (preventive or causative) of early hepatocarcinogenesis [23].

In the present study, DEN-initiated and CCl_4_-promoted protocols in males showed marked advantage to the classical DEN-initiated and non-promoted protocol, considering that only 33% to 78% mice submitted to the non-promoted method developed AHFs within 22 weeks, in a strain-dependent manner [46] (Table 3). Moreover, in the same model, a long time of experiment (28 weeks) was required for HCA development in 90% to 100% of male mice [9] in comparison to the present medium-term bioassay (Table 3). The current findings explicit the importance of fibrotic microenvironment induced by chronic CCl_4_ regimen on the emerging of hepatocellular preneoplastic and neoplastic lesions.

**Table 3:** Advantages of the proposed fibrosis-associated hepatocarcinogenesis models in males and females.

Models	Methods			Fibrosis	Incidence of end-point lesion	
	DEN	Promoter	Strain		AHF	HCA
***Males***						
*Proposed models*	*Single 10 mg/Kg (week 2)*	*CCl*_*4*_ *(3x/week; 0*.*25 to 1*.*50 μL/g*, *for 8 weeks)*	*C3H/HeJ*	*Yes*	*100% (week 17)*	*100% (week 17)*
	*Single 50 mg/Kg (week 2)*	*CCl*_*4*_ *(3x/week; 0*.*25 to 1*.*50 μL/g*, *for 8 weeks)*	*C3H/HeJ*	*Yes*	*100% (week 17)*	*100% (week 17)*
Chappell *et al*. (2014) [26]	Single 1 mg/Kg (week 2)	CCl_4_ (2x/week; 0.20 μL/g, for 14 weeks)	B6C3F1	Yes	31% (week 22)	100% (week 22)
Uehara *et al*. (2010) [47]	Single 1 mg/Kg (week 2)	CCl_4_ (2x/week; 0.20 μL/g, for 9 weeks)	B6C3F1	Yes	100% (week 17)	40% (week 17)
Goldsworthy & Fransson-Steen (2002) [46]	Single 1 mg/Kg (week 2)	Phenobarbital (500 ppm, for ~15–16 weeks)	C3H/HeJ	No	56% (week 22)	-
			B6C3F1	No	44% (week 22)	-
			C57BL	No	25% (week 22)	-
		None	C3H/HeJ	No	78% (week 22)	-
			B6C3F1	No	44% (week 22)	-
			C57BL	No	33% (week 22)	-
Weghorst *et al*. (1989)[9]	Single 5 mg/Kg (week 2)	Phenobarbital (500 ppm, for 24 weeks)	C3H/HeJ	No	-	100% (week 28)
			B6C3F1	No	-	100% (week 28)
			C57BL	No	-	50% (week 28)
		None	C3H/HeJ	No	-	100% (week 28)
			B6C3F1	No	-	100% (week 28)
			C57BL	No	-	90% (week 28)
***Females***						
*Proposed models*	*Single 10 mg/Kg (week 2)*	*CCl*_*4*_ *(3x/week; 0*.*25 to 1*.*50 μL/g*, *for 8 weeks)*	*C3H/HeJ*	*Yes*	*100% (week 17)*	*62*.*5% (week 17)*
	*Single 50 mg/Kg (week 2)*	*CCl*_*4*_ *(3x/week; 0*.*25 to 1*.*50 μL/g*, *for 8 weeks)*	*C3H/HeJ*	*Yes*	*100% (week 17)*	*25% (week 17)*
Romualdo *et al*. (2017) [12]	Single 50 mg/Kg (week 2)	Hexachlorobenzene (200 ppm, for 20 weeks)	Balb/C	No	100% (week 26)	-
Fukumasu *et al*. (2006) [53]	Single 10 mg/Kg (week 2)	None	Balb/C	No	100% (week 29)	-

DEN = diethylnitrosamine. “-” = data not available.

The broadly-applied DEN-initiated and phenobarbital (PB)-promoted protocol also proved to be less effective for AHF and HCA development in comparison to our male mice models. Under DEN/PB protocol, 25% to 56% of mice developed AHFs within 22 weeks and 50% to 100% displayed HCAs in 28 weeks, in a strain-dependent manner [9,46] (Table 3). In the light of the fact that PB is a non-fibrogenic promoter (Table 3) (*i*.*e*., not resembling the fibrotic background of 70% to 90% human HCC cases), DEN-initiated and CCl_4_-promoted protocols present clear advantage and thus, relevance to the study of the corresponding human disease.

Ultimately, our protocols for males increased in 60% the incidence of HCAs compared to other recent and similar fibrosis-associated hepatocarcinogenesis models in the intermediate-susceptible B6C3F1 strain [26,47] at the same time-point (100% *vs*. 40%) (Table 3). Its worthy of note that at week 17, these models showed non-significant incidence of HCC (20% of mice) as well as our DEN 10/CCl_4_ assay (12.5% of mice). In these studies, 22 weeks of experiment were necessary for all mice to develop HCAs (Table 3). In addition, these assays only presented incidence data on preneoplastic and neoplastic lesions, lacking on other important and commonly applied histopathological parameters as number of foci/cm^2^, size of foci, multiplicity of adenomas and others. All these parameters are presented and detailed in our models. Our results can be mostly attributed to the high-susceptible C3H/HeJ mice used herein. This specific strain displays genetic predisposition to hepatocarcinogenesis, developing HCC in senescence, independently of chemically-induced protocols [48]. Indeed, many studies have mapped multiple loci that can potentially confer hepatocarcinogenesis predisposition to this mouse strain [49,50].

In literature, chemically-induced models for hepatocarcinogenesis harbor a clear male predominance due to sex bias, reflecting the corresponding human disease [7]. In fact, experimental studies present solid evidence concerning the estrogen axis as a protective pathway for female hepatocarcinogenesis [7,51]. In accordance to previous sex bias data, our female models showed no IL-6 axis activation, decreased size and number of AHF and multiplicity of HCA compared to males, one week after the last CCl_4_ insult. However, as a novelty of our work, we proposed standard medium-term fibrosis associated-hepatocarcinogenesis female models (Table 3). In the current bioassays (DEN10/CCl_4_ and DEN50/CCl_4_), it was observed marked liver fibrosis, featuring collagen accumulation (Sirius red) and HSCs activation (α-SMA). In addition, females clearly showed diminished activity of antioxidant agents (catalase and glutathione system). As well as males, females presented enhanced apoptosis and cell proliferation in surrounding liver tissue. At the same time-point as males (week 17), both female protocols increased the incidence of preneoplastic AHFs and only DEN10/CCl_4_ significantly enhanced HCA incidence compared to vehicle group. Due to increased experimental time for neoplastic lesion development in females, most of bioassays present preneoplastic AHFs as end-point lesions [12,52]. In comparison to a classical DEN-initiated model in Balb/C mice, in the absence of a promoter stimulus, the models presented here diminished in twelve weeks the required experimental time for AHF development in all mice [52] (Table 3). Similar reduction can be observed when models are compared to DEN-initiated and hexachlorobenzene (HCB)-promoted model in Balb/C female mice [12] (Table 3) In addition to the long experimental time, the referred models lack liver fibrosis and do not apply HCAs as end-point lesions.

Although we presented optimized male and female fibrosis-associated hepatocarcinogenesis models, results indicate a threshold in the proposed DEN doses, not presenting a dose-response relationship for preneoplastic and neoplastic lesion development as in other mice models [53,54]. DEN 50/CCl_4_ protocol in males resulted in a lower HCA multiplicity, decreased AHF size, occupied area and proliferation whereas females presented non-significant incidence of HCAs in comparison to respective DEN10/CCl_4_ regimens. Besides, DEN 50-treated males exhibited marked features of toxicity including diminished food consumption and body weight from DEN initiation (week 2) until the end of the experiment (week 17). In adult rat, DEN-initiation exhibited a plateau as increased doses of DEN (>160 mg/Kg) did not necessarily lead to an increased number of liver GST-P^+^ AHF [55]. DEN initiation at high doses could have led to hepatocyte death instead of initiating these cells to hepatocarcinogenesis and, ultimately, resulting in decreased number of preneoplastic lesions [55]. In fact, we observed high liver damage in both male and female submitted to DEN 50 when compared to DEN 10, twenty-four hours after a single DEN administration (S6 and S7 Figs).

In order to evaluate the effect of DEN-initiation in the models, the authors performed a short-term study: male and female C3H/HeJ mice (n = 5 mice/sex/group) received single injections of diethylnitrosamine or saline vehicle (as described in item 2.1) at PND 14 and were euthanized a day after DEN-administration. HE-stained liver sections revealed that DEN at 50 mg/Kg increased the incidence of early bridging centrilobular necrosis compared to vehicle and DEN 10 mg-treated mice in both male (DEN 50: 100% *vs*. DEN 10 and Saline: 0%, p = 0.018, for both) and female (DEN 50: 100% *vs*. DEN 10: 20% and Saline: 0%, p = 0.048 and p = 0.018, respectively) (S6 and S7 Figs). Indeed, the occurrence of bridging necrosis in DEN 50 mg/Kg-treated mice, as observed in our short-term study, could have impaired preneoplastic and/or neoplastic lesion development in our male and female DEN 50/CCl_4_ models. However, the effect of intermediate doses (20, 30, 40 mg/Kg, for example) on preneoplastic and neoplastic lesion development should be evaluated in neonatal C3H/HeJ mice model in further studies.

In summary, we proposed standard fibrosis-associated early hepatocarcinogenesis models for male and female C3H/HeJ mice, applying a chemically-induced protocol in a high-susceptible mouse strain. As expected, these models showed clear sex bias and for DEN 10/CCl_4_ protocols in males and females, we reduced the required experimental time for the development of multiple preneoplastic (AHF) and neoplastic (HCAs) lesions in comparison to other classical mice models.

## Supporting information

S1 File NC3Rs ARRIVE guidelines checklist. (PDF)

S1 Fig Effects of different DEN/CCl_4_ protocols on body weight evolution of male and female C3H/HeJ mice. Dots are mean ± S.D. n = 8 mice/sex/group. DEN 10 or 50 = diethylnitrosamine 10 or 50 mg/kg b.wt. in 0.9% saline at week 2, respectively and CCl_4_ = carbon tetrachloride, i.p., 0.25 to 1.50 μL/g b.wt. in 10% corn oil solution for 8 weeks (see Material and methods section). *Statistical difference among groups by ANOVA and *post hoc* Tukey’s test (p<0.05).
(TIFF)

S2 Fig Representative photomicrographs of H&E-stained and immunostained (Ki-67) sections of hepatocellular adenoma (HCA) and hepatocellular carcinoma (HCC) found in the liver of DEN/CCl_4_-treated mice. (A, B, C) HCAs displayed typical loss of normal lobular architecture, enlarged cells, and vacuoles. (D, E, F) HCCs were composed of well-differentiated hepatocytes arranged in trabeculae of multiple cell layers and acinar structures, (A, D: 4× objective; scale bar = 200 μm) (B, C, E, F: 40× objective; scale bar = 20 μm).
(TIF)

S3 Fig Effects of different DEN/CCl_4_ protocols on β-catenin protein expression in male and female C3H/HeJ mice. (A) Representative photomicrographs β-catenin immunostained sections of surrounding liver tissue (40× objective; scale bar = 20 μm) and (B) preneoplastic and neoplastic lesions (10× objective, scale bar = 100 μm). Representative western blot bands and semiquantitative analysis of (C) male and (F) female mice. Values are mean + S.D. n = 6 mice/group. DEN 10 or 50 = diethylnitrosamine 10 or 50 mg/kg b.wt. in 0.9% saline at week 2, respectively and CCl_4_ = carbon tetrachloride, i.p., 0.25 to 1.50 μL/g b.wt. in 10% corn oil solution for 8 weeks (see Material and methods section).
(TIFF)

S4 Fig TGF-α expression in DEN/CCl_4_ models. (A) Representative photomicrographs of TGF-α immunostained sections of surrounding liver tissue (40× objective; scale bar = 20 μm) and (B) H&E-stained and respective TGF-α immunostained sections of basophilic foci and hepatocellular adenoma (20× objective; scale bar = 50 μm). DEN 10 or 50 = diethylnitrosamine 10 or 50 mg/kg b.wt. in 0.9% saline at week 2, respectively and CCl_4_ = carbon tetrachloride, i.p., 0.25 to 1.50 μL/g b.wt. in 10% corn oil solution for 8 weeks (see Material and methods section).
(TIFF)

S5 Fig Sex comparison of (A) transaminases data and (B) preneoplastic and neoplastic lesions in DEN 10 and DEN 50/CCl_4_-induced fibrosis-associated hepatocarcinogenesis models. Values are mean + S.D or box and whiskers. n = 8 mice/sex/group. DEN 10 or 50 = diethylnitrosamine 10 or 50 mg/kg b.wt. in 0.9% saline at week 2, respectively and CCl_4_ = carbon tetrachloride, i.p., 0.25 to 1.50 μL/g b.wt. in 10% corn oil solution for 8 weeks (see Material and methods section). ALT = alanine aminotransferase; AST = aspartate aminotransferase; AHF = altered hepatocyte foci; HCA = hepatocellular adenoma. Data were analyzed by Student t test (p<0.05).
(TIFF)

S6 Fig Representative photomicrographs of the liver of vehicle, DEN 10 and DEN 50-treated neonatal C3H/HeJ male mice. Above: scale bar = 100 μm, bellow: scale bar = 50 μm. In special, DEN 50-treated mice displayed early bridging cetrilobular necrosis, with swelling and diffuse inflammatory cell infiltrate. DEN 10 or 50 = diethylnitrosamine 10 or 50 mg/kg b.wt. in 0.9% saline at week 2. Mice were euthanized 24h after DEN injection.
(TIF)

S7 Fig Representative photomicrographs of the liver of vehicle, DEN 10 and DEN 50-treated nenonatal C3H/HeJ female mice. Above: scale bar = 100 μm, bellow: scale bar = 50 μm. In special, DEN 50-treated mice displayed early bridging cetrilobular necrosis, with swelling and diffuse inflammatory cell infiltrate. DEN 10 or 50 = diethylnitrosamine 10 or 50 mg/kg b.wt. in 0.9% saline at week 2. Mice were euthanized 24h after DEN injection.
(TIF)
