# Supplementary figures and images for: Fibrosis-associated hepatocarcinogenesis revisited: Establishing standard medium-term chemically-induced male and female models
Source: PLoS One. 2018 Sep 13;13(9):e0203879. doi: 10.1371/journal.pone.0203879 (PMC6136798; doi:10.1371/journal.pone.0203879)

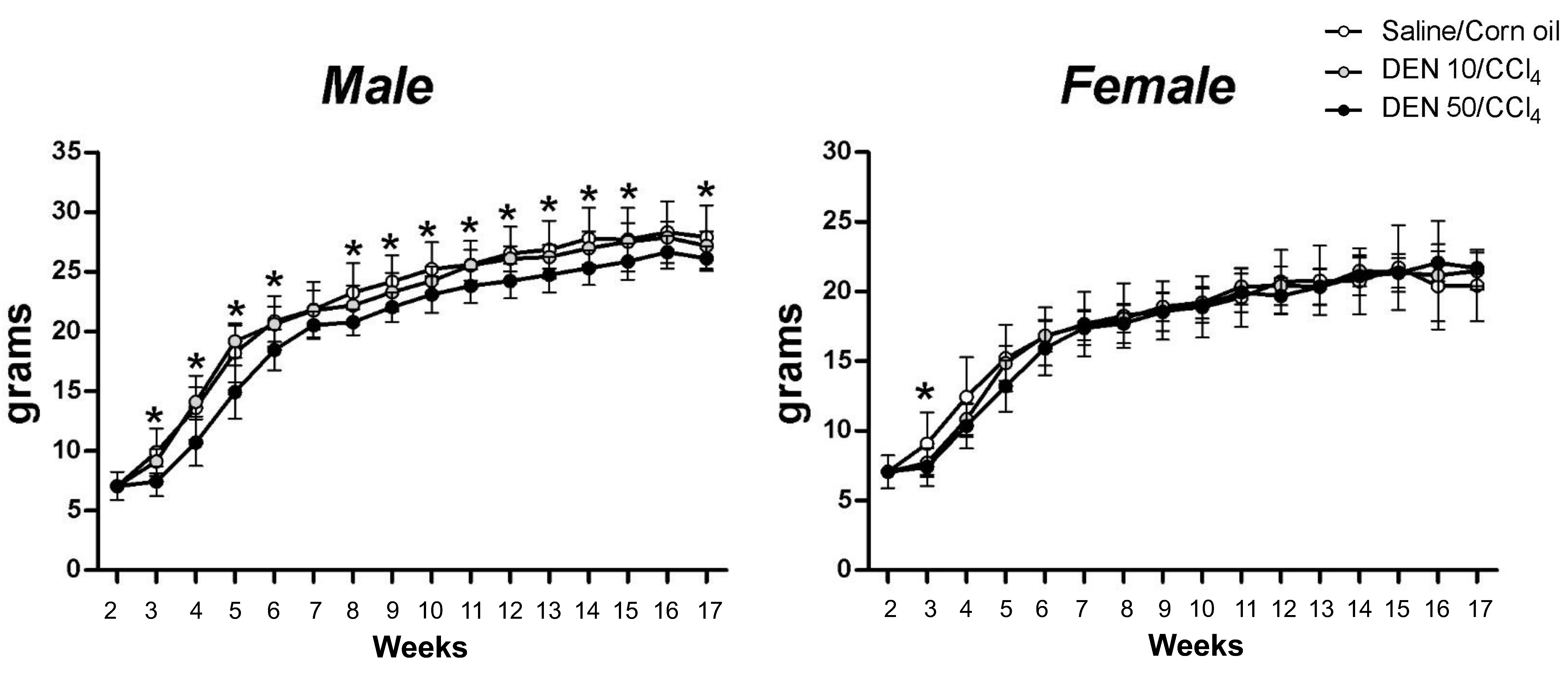

Supplement: S1 Fig — Dots are mean ± S.D. n = 8 mice/sex/group. DEN 10 or 50 = diethylnitrosamine 10 or 50 mg/kg b.wt. in 0.9% saline at week 2, respectively and CCl4 = carbon tetrachloride, i.p., 0.25 to 1.50 μL/g b.wt. in 10% corn oil solution for 8 weeks (see Material and methods section). *Statistical difference among groups by ANOVA and post hoc Tukey’s test (p<0.05). (TIFF) [file pone.0203879.s002.tiff]

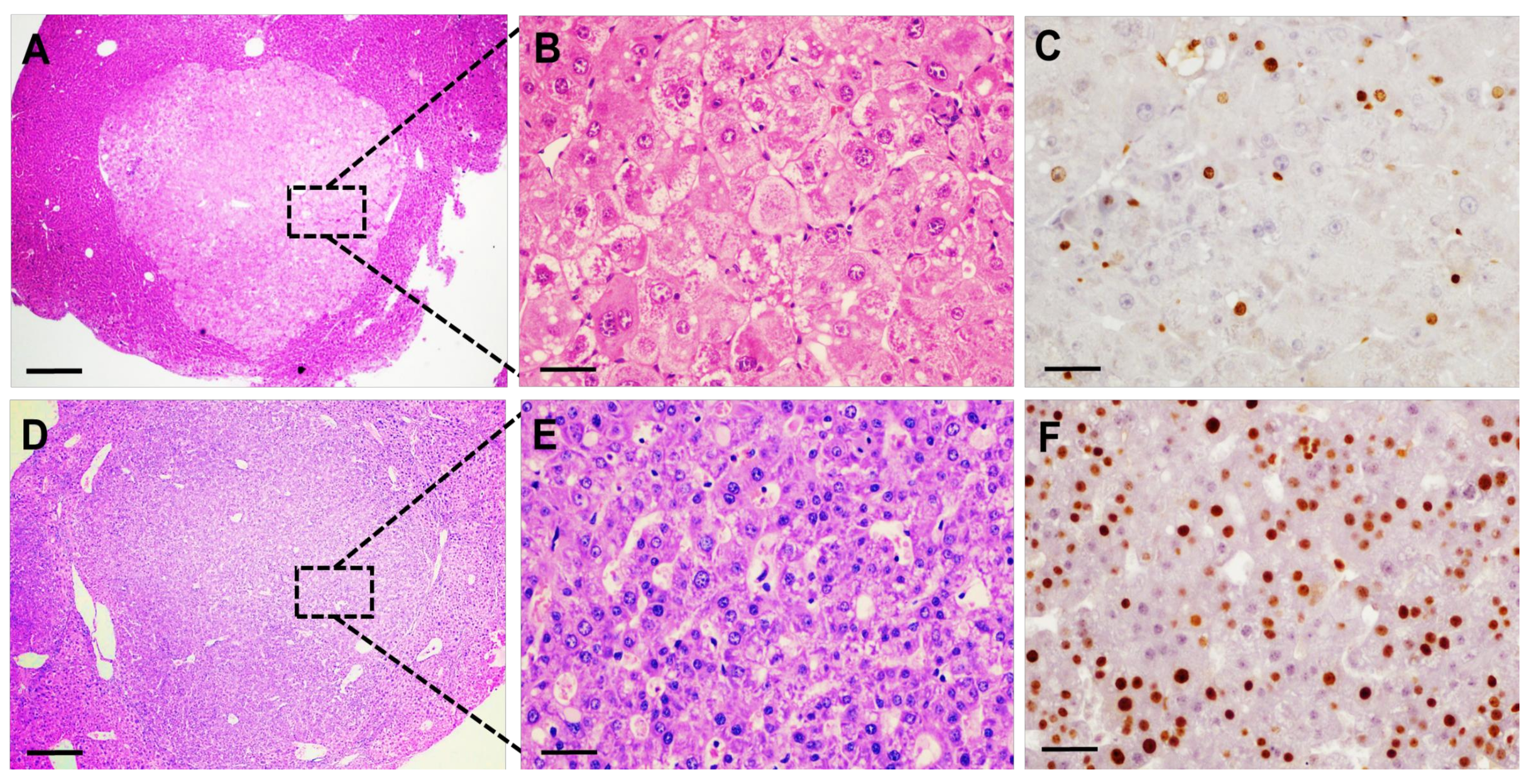

Supplement: S2 Fig — (A, B, C) HCAs displayed typical loss of normal lobular architecture, enlarged cells, and vacuoles. (D, E, F) HCCs were composed of well-differentiated hepatocytes arranged in trabeculae of multiple cell layers and acinar structures, (A, D: 4× objective; scale bar = 200 μm) (B, C, E, F: 40× objective; scale bar = 20 μm). (TIF) [file pone.0203879.s003.tif]

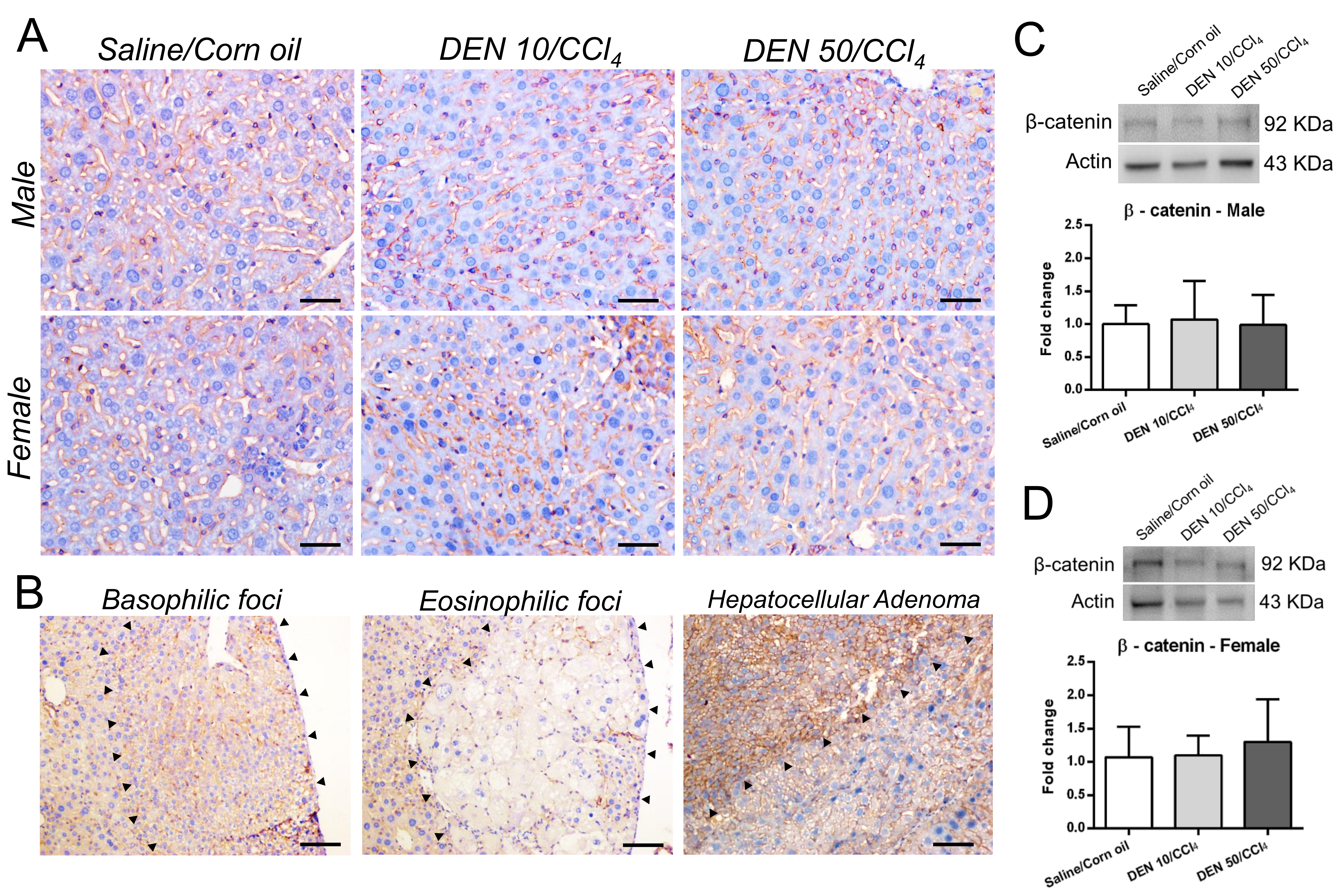

Supplement: S3 Fig — (A) Representative photomicrographs β-catenin immunostained sections of surrounding liver tissue (40× objective; scale bar = 20 μm) and (B) preneoplastic and neoplastic lesions (10× objective, scale bar = 100 μm). Representative western blot bands and semiquantitative analysis of (C) male and (F) female mice. Values are mean + S.D. n = 6 mice/group. DEN 10 or 50 = diethylnitrosamine 10 or 50 mg/kg b.wt. in 0.9% saline at week 2, respectively and CCl4 = carbon tetrachloride, i.p., 0.25 to 1.50 μL/g b.wt. in 10% corn oil solution for 8 weeks (see Material and methods section). (TIFF) [file pone.0203879.s004.tiff]

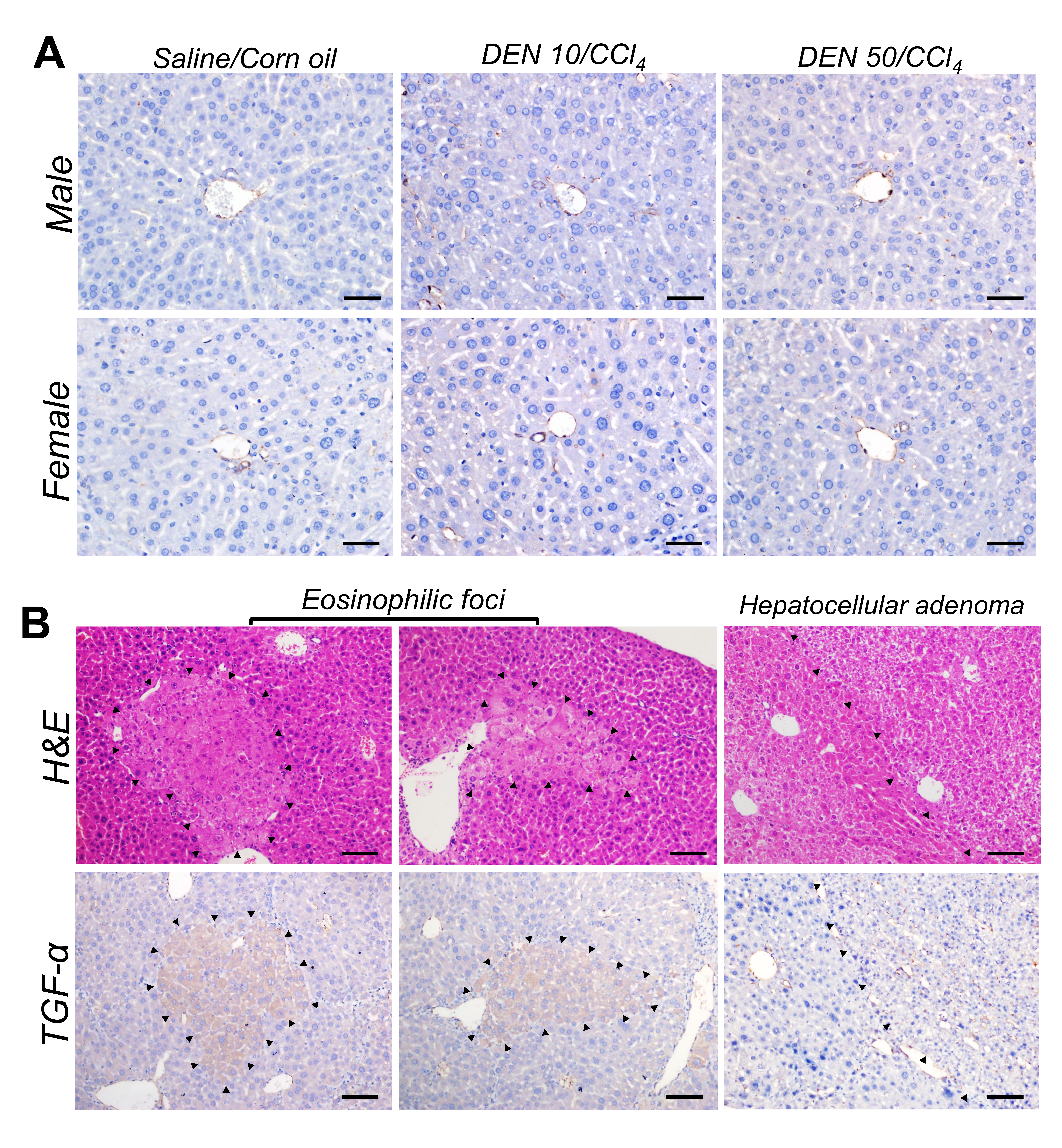

Supplement: S4 Fig — (A) Representative photomicrographs of TGF-α immunostained sections of surrounding liver tissue (40× objective; scale bar = 20 μm) and (B) H&E-stained and respective TGF-α immunostained sections of basophilic foci and hepatocellular adenoma (20× objective; scale bar = 50 μm). DEN 10 or 50 = diethylnitrosamine 10 or 50 mg/kg b.wt. in 0.9% saline at week 2, respectively and CCl4 = carbon tetrachloride, i.p., 0.25 to 1.50 μL/g b.wt. in 10% corn oil solution for 8 weeks (see Material and methods section). (TIFF) [file pone.0203879.s005.tiff]

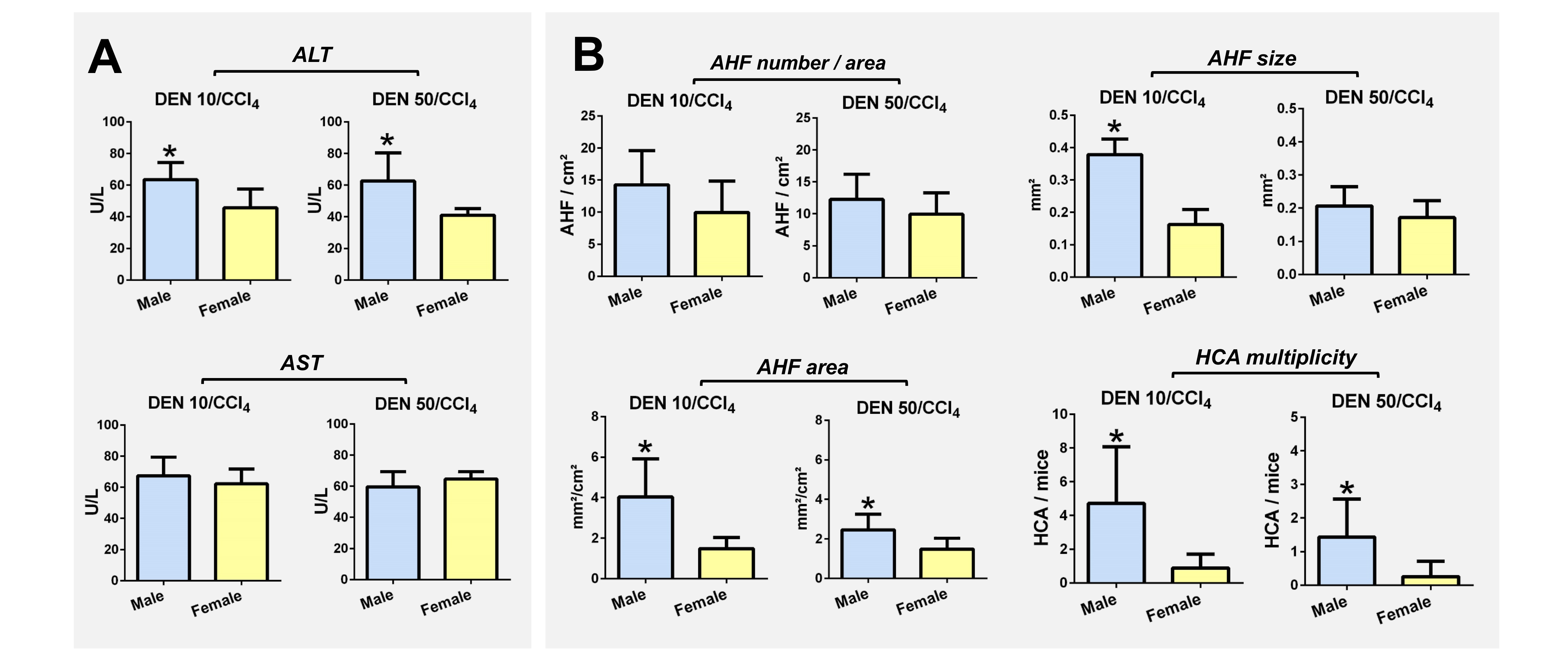

Supplement: S5 Fig — Values are mean + S.D or box and whiskers. n = 8 mice/sex/group. DEN 10 or 50 = diethylnitrosamine 10 or 50 mg/kg b.wt. in 0.9% saline at week 2, respectively and CCl4 = carbon tetrachloride, i.p., 0.25 to 1.50 μL/g b.wt. in 10% corn oil solution for 8 weeks (see Material and methods section). ALT = alanine aminotransferase; AST = aspartate aminotransferase; AHF = altered hepatocyte foci; HCA = hepatocellular adenoma. Data were analyzed by Student t test (p<0.05). (TIFF) [file pone.0203879.s006.tiff]

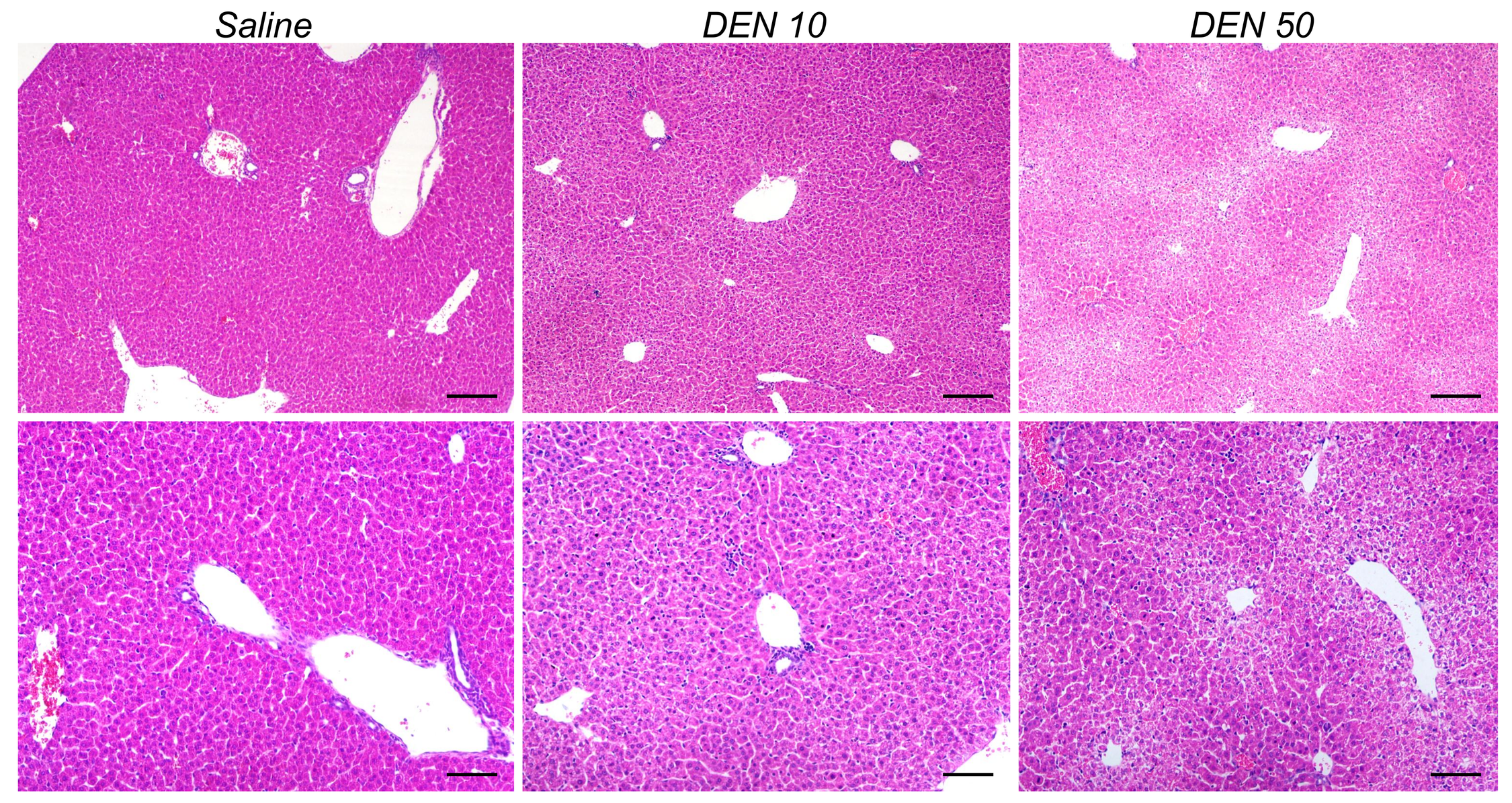

Supplement: S6 Fig — Above: scale bar = 100 μm, bellow: scale bar = 50 μm. In special, DEN 50-treated mice displayed early bridging cetrilobular necrosis, with swelling and diffuse inflammatory cell infiltrate. DEN 10 or 50 = diethylnitrosamine 10 or 50 mg/kg b.wt. in 0.9% saline at week 2. Mice were euthanized 24h after DEN injection. (TIF) [file pone.0203879.s007.tif]

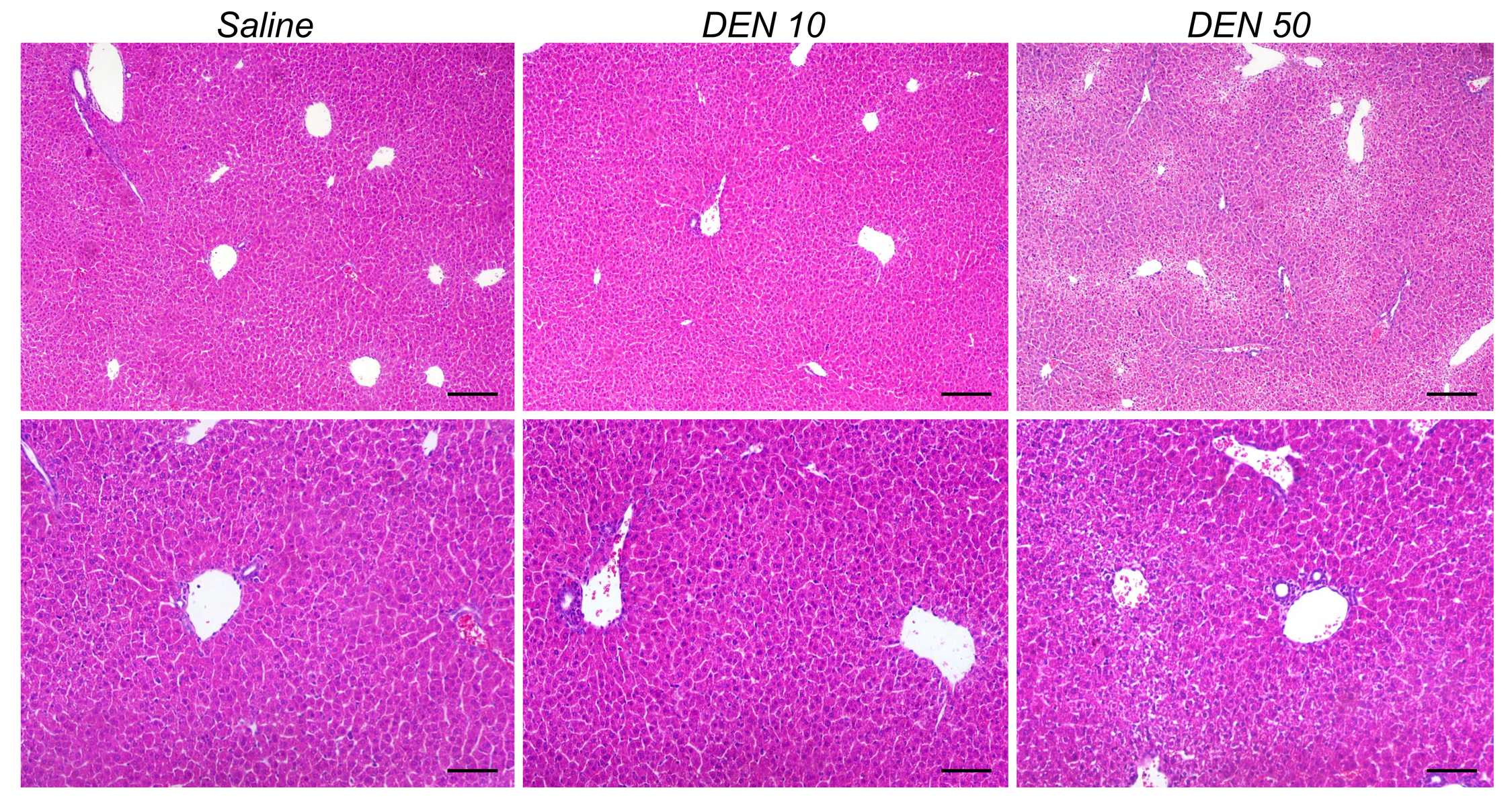

Supplement: S7 Fig — Above: scale bar = 100 μm, bellow: scale bar = 50 μm. In special, DEN 50-treated mice displayed early bridging cetrilobular necrosis, with swelling and diffuse inflammatory cell infiltrate. DEN 10 or 50 = diethylnitrosamine 10 or 50 mg/kg b.wt. in 0.9% saline at week 2. Mice were euthanized 24h after DEN injection. (TIF) [file pone.0203879.s008.tif]
